# Identification of the Plant Family Caryophyllaceae in Korea Using DNA Barcoding

**DOI:** 10.3390/plants12102060

**Published:** 2023-05-22

**Authors:** Dong-Pil Jin, Sunhee Sim, Jong-Won Park, Ji-Eun Choi, Jiwon Yoon, Chae Eun Lim, Min-Ha Kim

**Affiliations:** 1 Urban Biodiversity Research Division, Sejong National Arboretum, Korean Arboretum and Gardens Institute, Sejong 30106, Republic of Korea; 2 Plant Resources Division, National Institute of Biological Resources, Incheon 22689, Republic of Korea; 3 Department of Biology Education, Chonnam National University, Gwangju 61186, Republic of Korea

**Keywords:** Caryophyllaceae, chloroplast DNA, DNA barcoding, internal transcribed spacer, plant resources

## Abstract

Caryophyllaceae is a large angiosperm family, with many species being utilized as ornamental or medicinal plants in Korea, in addition to several endangered species that are managed by the government. In this study, we used DNA barcoding for the accurate identification of Korean Caryophyllaceae. A total of 78 taxa (*n* = 215) were sequenced based on three chloroplast regions (*rbc*L, *mat*K, and *psb*A–*trn*H) and nuclear ribosomal internal transcribed spacers (ITS). In the neighbor-joining tree, a higher accuracy of identification was generally observed when using ITS (>73%) rather than chloroplast regions (<62%). The highest resolution was found for *rbc*L + ITS (77.6%), although resolution varied according to the genus. Among the genera that included two and more species, five genera (*Eremogone*, *Minuartia*, *Pseudostellaria*, *Sagina*, and *Stellaria*) were successfully identified. However, the species of five other genera (*Cerastium*, *Gypsophila*, *Dianthus*, *Silene*, and *Spergularia*) showed relatively low resolutions (0–61.1%). In the cases of *Cerastium*, *Dianthus*, and *Silene*, ambiguous taxonomic relationships among unidentified species may have been a factor contributing to such low resolutions. However, in contrast to these results, *Gypsophila* and *Spergularia* have been identified well in previous studies. Our findings indicate the need of taxonomic reconsideration in Korea.

## 1. Introduction

Caryophyllaceae is a large family of angiosperms that includes approximately 2200 species across 86 genera, with the majority of plants in the family being found across the Northern Hemisphere [1]. Many members of Caryophyllaceae are commonly planted in gardens (e.g., *Dianthus* L. (carnation) and *Gypsophila* L. (baby’s-breath)) or are used as medicinal resources (e.g., *Cerastium* L. (mouse-ear chickweed), *Silene* L. (catchfly), and *Stellaria* L. (chickweed), summarized in [2]). In Korea, 66–91 taxa of 17 genera are recognized, including alien plants [3,4], and these taxa have various uses, similarly to other Caryophyllaceae species. For example, compounds of *Gypsophila oldhamiana* Miq. (having cytotoxicity against tumor cell), *Silene firma* Siebold & Zucc. (used for anuria, breast cancer, gonorrhea, and diseases of lactation), *Stellaria aquatica* (L.) Scope. (applied to pneumonia and high blood pressure), and *Pseudostellaria heterophylla* (Miq.) Pax (applied to tuberculosis, physical fatigue) have been investigated for their use as medicinal resources [5,6,7,8]. Additionally, to improve the diversity of ornamental crops and farm income, native Korean plants, such as *Minuartia laricina* (L.) Mattf. and *Arenaria juncea* M. Bieb., were tested and distributed under the national government’s plan [9]. Furthermore, *Lychnis kiusiana* Makino, *Lychnis wilfordii* (Regel) Maxim., and *Silene capitata* Kom. have been designated as endangered species II by the government [10]. Moreover, several other species are regarded as weeds of agricultural land [11,12], and alien plants of the family have been previously reported [13,14,15,16,17]. Such weeds and alien plants could lead to a decrease in crop yields and the disturbance of native species [18,19]. As a result, endangered, weed, and alien plants must all be continuously monitored; therefore, the precise identification of Caryophyllaceae species is critical for the use of resources and the management of weeds and alien species. However, factors such as morphological variations and polyploidy [20,21], and their commercial products being often sold as plant parts, tea bags, or powders, hinder accurate species identification and may even result in the misuse of resource species or a decrease in product quality.

For accurate and rapid identification of species, numerous previous studies have utilized molecular tools. In particular, DNA barcoding has been the primary method used for the last two decades. DNA barcoding identifies species using short nucleotide sequences or “barcodes” that can be applied across a variety of species [22,23]. In animal models, the cytochrome c oxidase I (CO1) gene is a commonly used DNA barcode [24,25]. However, its use as a DNA barcode in plants is difficult due to its slow and uneven mutation rate. Instead, two genes (*rbc*L and *mat*K) of chloroplasts were suggested as core barcodes for plant identification. Generally, *rbc*L shows a high success rate of polymerase chain reaction (PCR) with relatively low resolution, whereas *mat*K exhibits a better resolution despite the relatively low success rate of PCR [26]. However, these genes often show weak discriminatory power for lower taxa [27]; therefore, internal transcribed spacers (ITS) of nuclear ribosomal DNA and non-coding regions (e.g., *psb*A–*trn*H, *atp*F–*atp*H, and *psb*K–*psb*I) of chloroplasts that have more variable sites are suggested as additional regions [28,29]. In practice, DNA barcoding has been applied to the management of plant product quality [30,31], species conservation [32], the control of alien plants [33,34], and exploration of regional flora [35].

With regards to taxonomy, three subfamilies have been recognized in Caryophyllaceae: Alsinoideae, Caryophylloideae, and Paronychioideae [1,36,37]. However, morphological homoplasy within Caryophyllaceae make circumscribing the subfamilies difficult [1,38,39]. Molecular phylogenetic studies of the family also disregarded monophyly of traditional subfamilies; alternatively, the tribal classification was applied [38,39,40]. Similarly, the polyphyly of many genera was detected based on molecular phylogeny, such as *Arenaria* L., *Gypsophila*, *Minuartia* L., and *Silene* [40]. Therefore, taxonomic revisions on the Caryophyllaceae taxa have been conducted in accordance with molecular results, e.g., [41,42,43,44].

In this study, we aimed to identify Caryophyllaceae species in Korea using DNA barcoding. Considering previous DNA barcode studies, four regions were investigated: two crucial DNA barcodes (*rbc*L and *mat*K) and two non-coding regions (*psb*A–*trn*H and ITS). In addition, information on nucleotide variation, PCR success rate, and discriminatory power was provided using a combination of barcodes. Those results would be utilized as reference of the molecular identification of Korean Caryophyllaceae. In addition, it has been concluded that sequences of various species can provide more detailed taxonomic relationships among taxa. Therefore, we believe that our data will contribute to the taxonomy and phylogeny of Caryophyllaceae.

## 2. Results

### 2.1. Characteristics of DNA Barcode Regions

**Figure 1:**
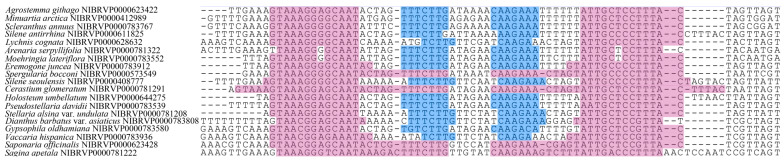
Alignment of *psb*A–*trn*H sequences in Korean Caryophyllaceae. Short inverted repeats are indicated with color-shaded rectangles (blue and pink). Each color-shaded rectangle matches with the same color-shaded rectangles of the opposite side.

The nucleotide sequences (*rbc*L, *mat*K, *psb*A–*trn*H, and ITS) of 78 taxa including two forma (215 individuals) in Caryophyllaceae from Korea were obtained, and the information for each region is presented in Table S1. In terms of PCR success rate, all analyzed individuals were well amplified (*rbc*L = 100%, *mat*K = 100%, *psb*A–*trn*H = 100%, and ITS = 100%). The sequencing success rate for all regions (*rbc*L, *mat*K, *psb*A–*trn*H, and ITS) reached 100%; however, direct sequencing of the *psb*A–*trn*H region was not always successful. These results were clear after reading the region, poly A/T, or short inverted repeat (Figure 1). In the case of ITS, multiple peaks were observed across genera, and direct sequencing of *Dianthus*, *Eremogone*, and *Stellaria* was occasionally limited. When both forward and reverse sequencing results showed multiple peaks at a certain point, we treated that position as a mixed base. Nucleotide sequences were aligned without indels in *rbc*L (689 bp), whilst length variations were observed in the other regions: *mat*K = 746–782 bp (aligned length = 784 bp), *psb*A–*trn*H = 160–404 bp (aligned length = 600 bp), and ITS = 606–637 bp (aligned length = 672 bp) (Table 1). The percentage of variable sites was highest in *psb*A–*trn*H (66.3%), followed by ITS (58.3%), *mat*K (55.0%), and *rbc*L (18.0%) (Table 1). This order was the same for the percentage of Parsimony informative sites: *psb*A–*trn*H (61.8%), ITS (56.5%), *mat*K (52.4%), and *rbc*L (16.7%) (Table 1).

### 2.2. Evaluation of DNA Barcodes

**Figure 2:**
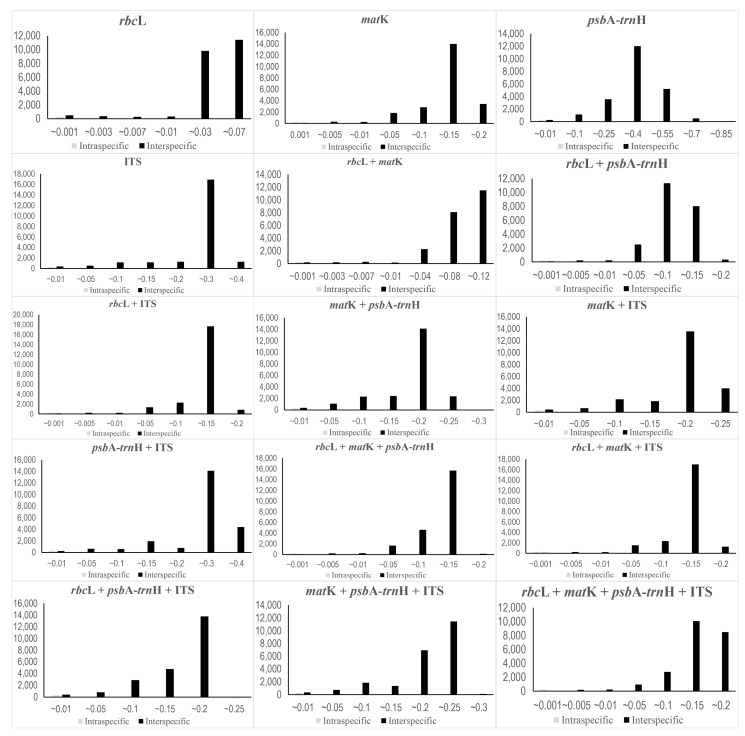
A test of barcode gap in Korean Caryophyllaceae based on variable barcode combinations. X-axes indicate Kimura 2-parameter (K2P) distances and y-axes represent the occurrences.

The mean interspecific pairwise distance was highest in *psb*A–*trn*H (0.3245), followed by ITS (0.2259), *mat*K (0.1140), and *rbc*L (0.0291) (Table 2). Furthermore, the mean intraspecific genetic distance was also the highest in *psb*A–*trn*H (0.0078), followed by ITS (0.0037), *mat*K (0.0019), and *rbc*L (0.0004) (Table 2). These values were 42–73 times larger in interspecific pairwise distances than in intraspecific pairwise distances. When combining the regions, the mean of interspecific pairwise distance is highest in *psb*A–*trn*H + ITS (0.2502), and the mean of intraspecific genetic distance is also highest (0.0048) (Table 2). However, an overlap was noted in genetic distance between intraspecific and interspecific pairwise distances for all regions (Figure 2); as a result, there was no distinct barcode gap.

**Figure 3:**
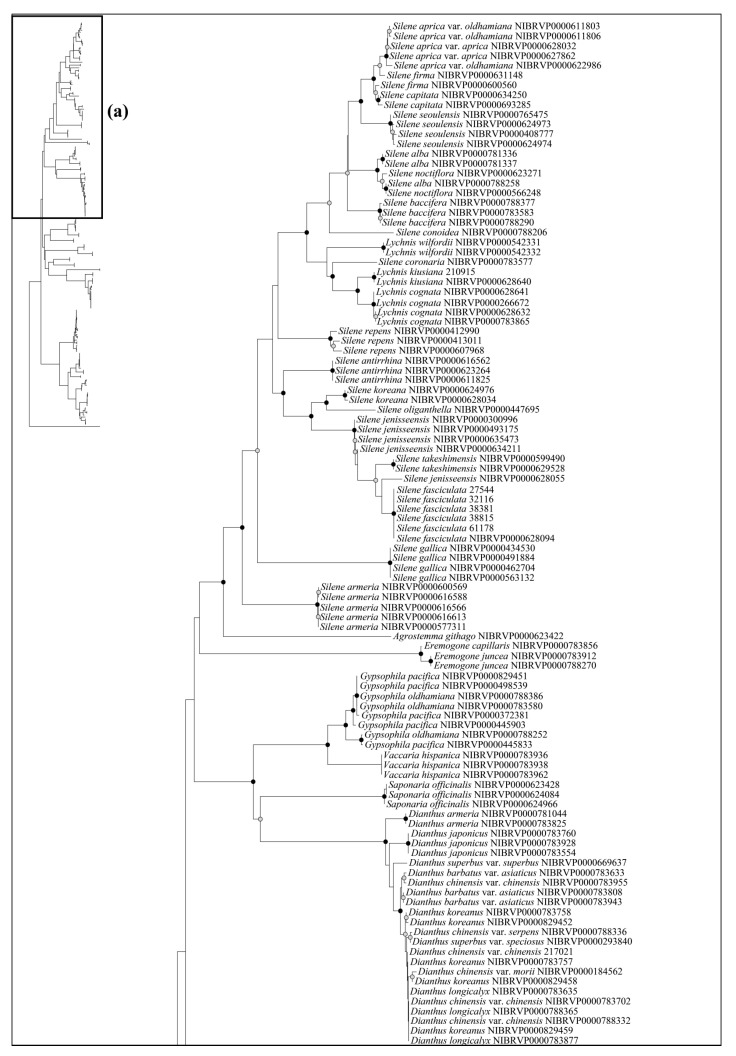

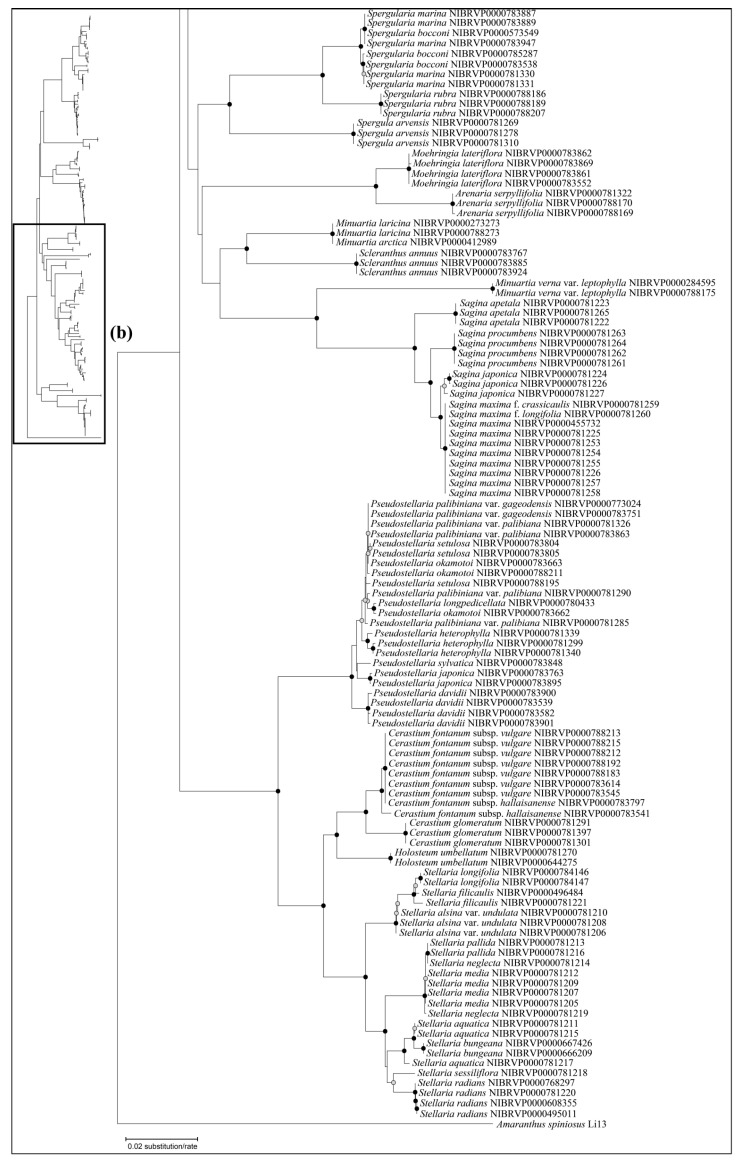
The neighbor-joining phylogenetic tree of the Korean Caryophyllaceae based on combined sequence from three chloroplast DNA regions (*rbc*L + *mat*K + *psb*A–*trn*H). This tree is split into (**a**,**b**). The schematic phylogenetic tree in the top left represents the combined topology (**a**,**b**). The circles on the nodes represent bootstrap values (black dot = >80%, gray dot = >50%).

**Figure 4:**
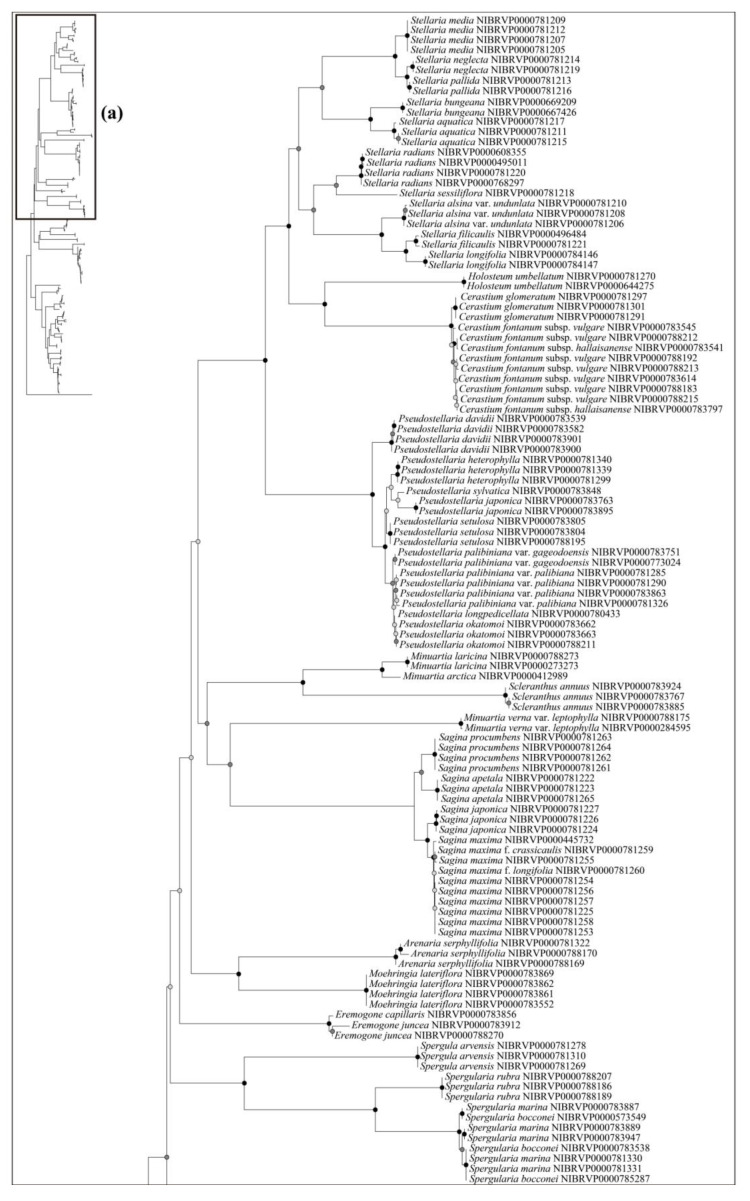

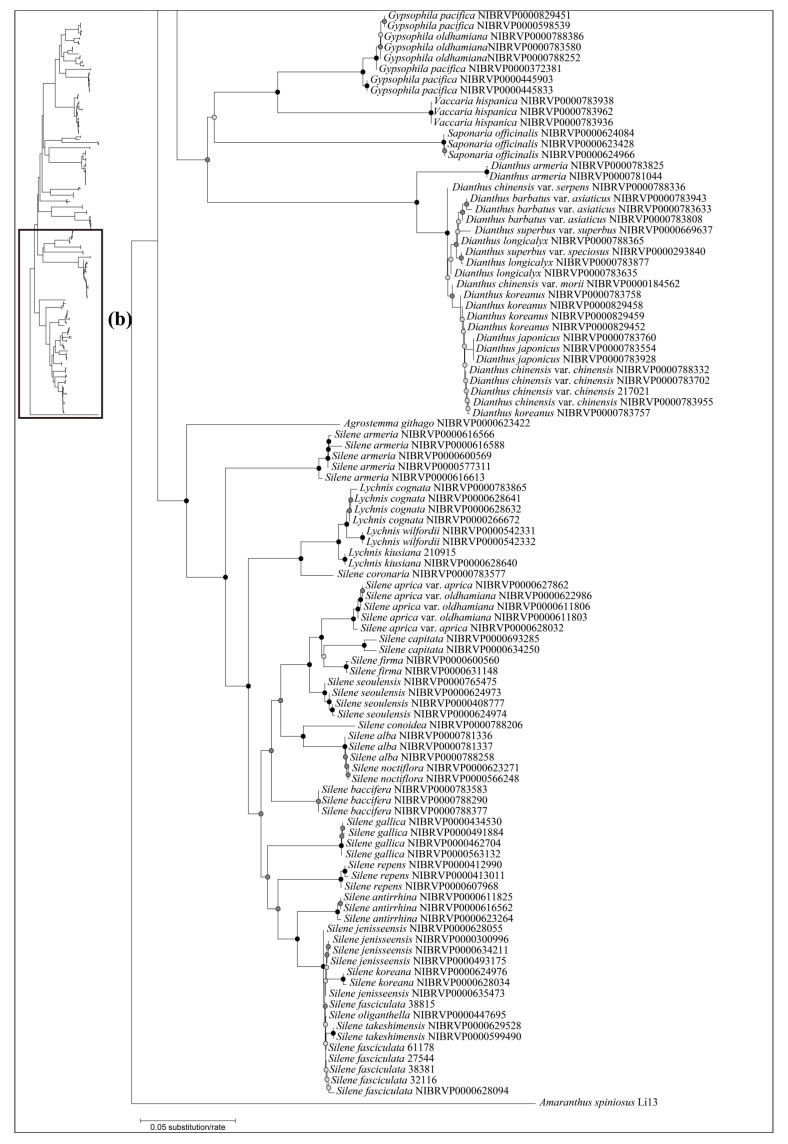
The neighbor-joining phylogenetic tree of the Korean Caryophyllaceae based on internal transcribed spacer. This tree is split into (**a**,**b**). The schematic phylogenetic tree in the top left represents the combined topology (**a**,**b**). The circles on the nodes represent bootstrap values (black dot = >80%, gray dot = >50%).

**Figure 5:**
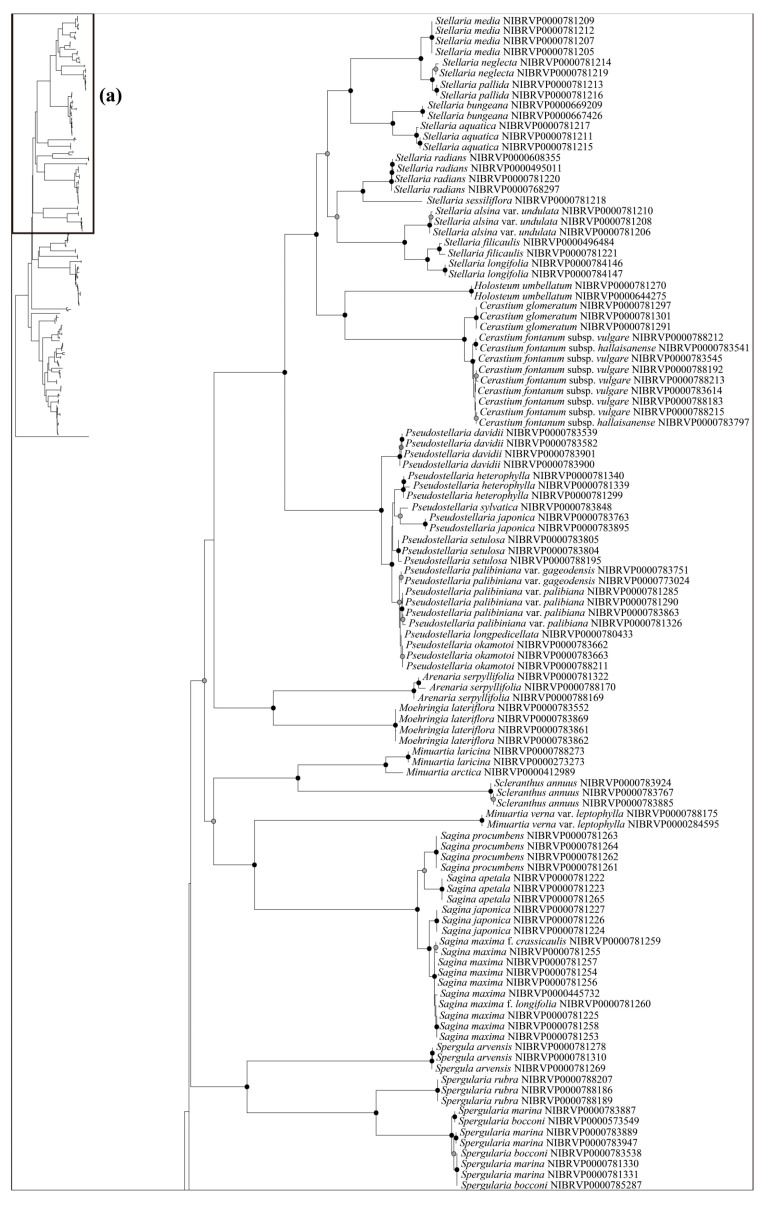

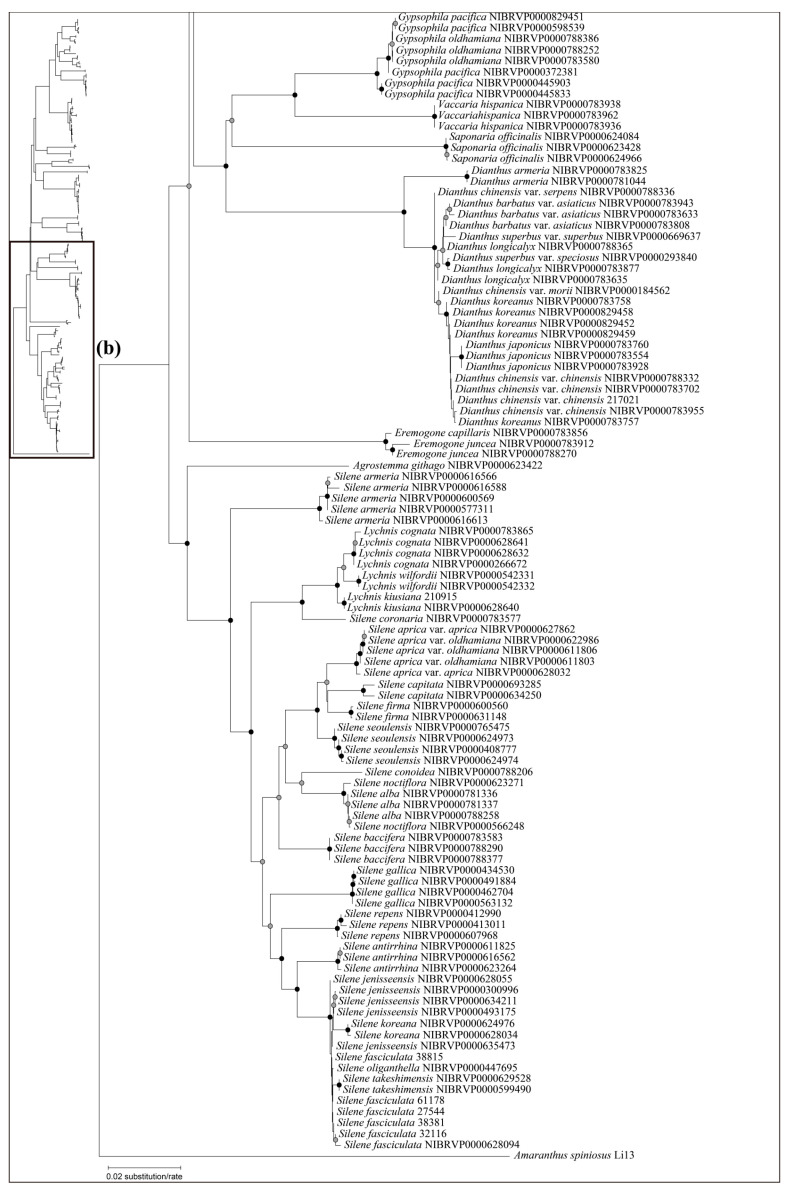
The neighbor-joining phylogenetic tree of the Korean Caryophyllaceae based on combined sequence (internal transcribed spacer + *rbc*L). This tree is split into (**a**,**b**). The schematic phylogenetic tree in the top left represents combined topology (**a**,**b**). The circles on the nodes represent bootstrap values (black dot = >80%, gray dot = >50%).

The ability to identify species using the DNA barcode regions was also evaluated using the phylogenetic analysis—specifically, the Neighbor-Joining (NJ) method (Figure 3, Figure 4 and Figure 5). Generally, a higher degree of species resolution was observed in cases using ITS (>73%) compared with those using only chloroplast regions (<62%) (Table 3); therefore, the best resolution was shown in ITS + *rbc*L (77.63%) (Figure 5). The best close match also showed similar results with phylogenetic analysis (Table 3). Overall, a higher success rate was observed in combined DNA barcode regions than a single region. Especially, cases using ITS (>69%) showed a higher degree of species identification (“correct” in Table 3) than those using only chloroplast regions (<68%). Differing with the phylogenetic analysis, using the combined four regions and ITS + *mat*K + *psb*A–*trn*H showed the best resolution (76.74%), while lower resolution was observed in ITS + *rbc*L (70.69%). As with the NJ and best close match analyses, better species partitions were observed in cases using combined regions compared to a single region. Under the best asap-score, the highest species partition was observed when using all regions (number of subsets, 40; asap-score, 8.00). In addition, the ASAP exhibited better resolution in cases using ITS compared to those using only chloroplast regions (Figures S1–S14).

Based on the best resolution tree (ITS + *rbc*L) (Figure 5), most genera were identified on all NJ trees, except for *Minuartia* and *Silene*. The ability for species identification varied according to the genus. Among the genera that included two or more taxa, all five genera (*Eremogone* Fenzl, *Minuartia*, *Pseudostellaria* Pax, *Sagina* L., and *Stellaria*) formed a clade, indicating successful identification (Figure 5). Each individual for *Eremogone capillaris* (Poir.) Fenzl and *Minuartia arctica* (Steven ex Ser.) Graebn. was investigated on the NJ tree; these were thought to have been distinguishable due to sufficient branch divergence from related species. In contrast, species of five genera (*Cerastium*, *Gypsophila*, *Dianthus*, *Silene*, and *Spergularia* (Pers.) J. Presl & C. Presl) were moderately or hardly distinguished at all (0–61.11%). Two species of *Gypsophila* (*Gypsophila oldhamiana* and *Gypsophila pacifica* Kom.) showed no separation from each other (0%), whilst *Cerastium* and *Spergularia* also showed low resolution (33.33%). *Cerastium glomeratum* Thuill. was shown to be separated from the two subspecies of *Cerastium fontanum* Baumg. (subsp. *hallaisanense* (Nakai) J.S. Kim and subsp. *vulgare* (Hartm.) Greuter & Burdet); however, these subspecies were not distinguishable from each other. Similarly, in *Spergularia*, only *Spergularia rubra* (L.) J. Presl & C. Presl could be identified from *Spergularia bocconei* (Scheele) Asch. & Graebn. and *Spergularia marina* (L.) Griseb. The DNA barcodes showed better discriminatory power for *Dianthus* (60.00%) and *Silene* (61.11%). In *Dianthus*, *Dianthus armeria* L., *Dianthus barbatus* L. var. *asiaticus* Nakai, and *Dianthus japonicus* Thunb. were all clustered in each clade. However, *Dianthus chinensis* var. *serpens* Y. N. Lee, *Dianthus chinensis* var. *morii* (Nakai) Y. C. Chu, *Dianthus superbus* L. var. *superbus*, and *Dianthus superbus* var. *speciosus* Rchb. diverged into a branch separate from other taxa and were sequenced based on only one individual. In addition, the majority of *Dianthus* species showed multiple types on the ITS; therefore, we concluded that these four taxa may not be distinguishable. In *Silene*, *Silene capitata* Kom., *Silene firma*, *Silene seoulensis* Nakai, *Silene baccifera* (L.) Roth, *Silene gallica* L., *Silene repens* Patrin ex Pers., *Silene antirrhina* L., *Silene koreana* Kom., and *Silene takesimensis* Uyeki & Sakata were all clustered into each clade according to morphological identification. Only one individual was sequenced in the case of *Silene conoidea* L., although this was clearly separated from the other. Despite *Silene aprica* Turcz. Ex Fisch. & C. A. Mey. being distinguishable at the species level, it was more difficult to distinguish at the intraspecies level.

## 3. Discussion

In this study, we examined four DNA barcoding regions in Korean Caryophyllaceae species. Ideal DNA barcodes allow for easy amplification and have sufficient variable sites to identify species [22]. Across taxa, all regions were well amplified with universal primers and sequences were obtained successfully. These results corresponded with the criteria for easy amplification. However, when considering their discriminatory ability, the combination of ITS was deemed as the optimal DNA barcode, with 71.43 to 75.32% in the phylogenetic analyses (Table 3). The best close match supported such a result of phylogenetic analysis, although there are some differences between the two analyses (Table 3). This identification resolution was comparable to that of other angiosperm taxa [45,46,47]. ITS is well known to have many informative sites; therefore, this region has previously been suggested as a DNA barcode in other plants because of its high resolution [47,48,49,50,51]. These results may have been influenced by the mutation rate of ITS being higher than that of chloroplast genes (*rbc*L and *mat*K) [52,53]. Similar to the results of previous studies, our results showed a higher discriminatory power when using ITS (>72%) than when using only chloroplast DNA regions (<61%) (Table 3). A low amplification rate of ITS has occasionally been reported according to the taxon [54], although such a problem was not observed in this study.

Although the addition of the *psb*A–*trn*H region (74.03%) did not improve the ability of species identification when compared with using only ITS (74.03%), this region did enhance discriminatory power at the genus level (Table 3). In the ITS and ITS + *rbc*L trees, *Silene oliganthella* Nakai ex Kitag. was not well distinguished from the congeneric species *Silene fasciculata* Nakai and *Silene jenisseensis* Willd. because of its short branch (Figure 4 and Figure 5). Meanwhile, *Silene oliganthella* diverged deeply from the latter with a long branch on the ITS + *psb*A–*trn*H tree (Figure S15). However, such expectations require caution, considering that only one sample of *Silene oliganthella* was included in the present study. In addition, with regard to taxonomy, the species was regarded as a variety or synonym of *Silene jenisseensis* [55]. Despite considerable sequence variation in *psb*A–*trn*H attributed to divergence among *Silene* species, simultaneous high variation within species disturbed precise identification in the case of *Pseudostellaria*. Sequence variations within species were detected, although some were shared with individuals of other species. Such a low resolution of *psb*A–*trn*H has also been observed in other lineages [56,57]. In addition, bi-directional sequencing of *psb*A–*trn*H often failed, which was thought to be due to long mononucleotide repeats [58,59] or a potential loop structure [60]. In fact, slippage after long mononucleotide repeats was observed in *Dianthus* and *Saponaria*, whilst a pair of inverted repeats that could form a cruciform shape was found in *Cerastium*, *Sagina*, and *Spergularia* (Figure 1). Therefore, in this study, we suggest ITS as a DNA barcode for Korean Caryophyllaceae species when considering both cost efficiency and discriminatory power, such as previous DNA barcoding studies [47,48,49,50,51]. Furthermore, using *rbc*L is recommended for better resolution (Table 3). In addition, *psb*A–*trn*H could be used as a supplementary region for the identification of a genus such as *Silene*. It is probable when considering the high degree of species identification in the best close match (Table 3). When considering cases where two DNA barcode regions were combined, it was supported by the ASAP result that the highest degree of species partitioning (37) was observed in ITS + *psb*A–*trn*H (under the best ASAP score).

Most of the genera in Korea were accurately identified in our phylogenetic tree, with the exception of *Minuartia* and *Silene*, which were identified as polyphyletic groups (Figure 5). However, this was not due to a lack of discriminatory power in the DNA regions used in this study. Molecular phylogenies have previously suggested that these two genera are polyphyletic [44,61]. In the case of *Minuartia*, the three species (*Minuartia arctica*, *Minuartia laricina,* and *Minuartia verna* (L.) Hiern var. *leptophylla* (Rchb.) Nakai) analyzed in this study each belong to different sections within the genus (summarized in [61]). The molecular phylogeny also showed that each of these species are included in different clades [61]. In fact, *Minuartia laricina* and *Minuartia verna* var. *leptophylla* were not analyzed in the analysis but their position in the phylogenetic tree could be inferred based on closely related taxa. As a result, they have been treated as *Cherleria arctica* (Steven ex Ser.) A. J. Moore & Dillenb., and *Pseudocherleria laricina* (L.) Dillenb. & Kadereit [61,62]. *Minuartia verna* var. *leptophylla* was not mentioned in the previous study [61]; it may be treated as a taxon of *Sabulina* Rchb. *Silene* has also been suggested as a polyphyletic group. In that study, *Silene armeria* L. (=*Atocion armeria* (L.) Raf.) were placed within the sister group of *Silene*. *Lychnis* species were posited within *Silene*; so, they were treated as a section of *Silene*. In light of the polyphyly of *Minuartia* and *Silene*, further investigation may be necessary to evaluate the validity of their current taxonomic classification in Korea.

Although most species of Caryophyllaceae were identified well using DNA barcodes, five genera (*Cerastium*, *Gypsophila*, *Dianthus*, *Silene*, and *Spergularia*) showed a low resolution (0–61.11%). Such a low resolution may have been caused by wide morphological variations within species and hybridization among species. In the case of *Cerastium*, subspecies of *Cerastium fontanum* (subsp. *hallaisanense* and subsp. *vulgare*) were not distinguished and were instead considered taxa [63]. Similar to *Cerastium*, unidentified *Silene* species in the NJ tree were thought to be undistinctive taxa (*Silene takesimensis*, *Silene fasciculata*, *Silene oliganthella*) [64]. For *Dianthus*, many species could have intercrossed both naturally and through cultivation (summarized in [65]). In reality, multiple peaks in the ITS region are frequently detected in the genus, which can make identification difficult. Therefore, the low rate of identification of these genera is not surprising considering the taxonomic similarities between them. In contrast, *Spergularia bocconei* could be distinguished from *Spergularia marina* by its dense glandular hair on stems and leaves [16], in addition to having smaller capsules/seeds than the latter [66]. *Gypsophila pacifica* and *Gypsophila oldhamiana* could also be distinguished by their leaf (ovate vs. oblong), inflorescence (diffuse vs. dense), and flower morphologies (pedicels, 2–5 mm vs. 5–10 mm; apex of petals, rounded vs. truncate or retuse; stamens and styles, shorter than petals vs. longer than petals) [3]. The *Spergularia* and *Gypsophila* species have been well identified using ITS2 and *trn*L–F [67], and ISSR and RAPD [68]. However, previous studies are in contrast to our results. There might be various reasons for such results, such as genetic variation across different regions or misconceptions about the taxonomic classification of these two genera. However, it is difficult to determine the exact cause based on the current study alone. Therefore, further investigation of morphological and genetic variations is necessary to better understand the relationships between these two genera in Korea.

## 4. Materials and Methods

### 4.1. Taxon Sampling

A total of 215 individuals representing 78 taxa across 17 genera were collected through field surveys, and dried specimens were collected from the herbarium of the National Institute of Biological Resources (NIBR). Leaves sampled from the field were immediately dried using silica gel. We attempted to cover the majority of species in Caryophyllaceae by citing the National List of Species of Korea [4]. For plants that grow in North Korea, we utilized samples collected from nearby regions, such as China and Russia. To represent genetic variation within species, we collected three or more samples per taxon from different populations, excluding plants that grow in North Korea. The information of samples is shown in Table S1.

### 4.2. DNA Extraction, Polymerase Chain Reaction (PCR), and Sequencing

Total genomic DNA of the abovementioned plants was extracted from silica-dried leaves using NucleoSpin^®^ Plant II (Macherey-Nagel, Düren, Germany), according to the manufacturer’s protocol, although the incubation times during cell lysis and elution of DNA were manually modified. The concentration of extracted DNA was subsequently measured using a Synergy LX microplate reader (BioTek Instruments, Winooski, VT, USA).

PCR was carried out to amplify DNA barcode regions, and each reaction mixture contained approximately 10 ng of DNA, 10 μL of AccuPower^®^ Taq PCR PreMix (Bioneer, Daejeon, Republic of Korea), distilled water, and appropriate volume of primers (usually 0.3 μM) in a total volume of 20 μL. The primers were selected with reference to previous studies so that they could be applied to the taxa of Caryophyllaceae (Table 4). The reaction was conducted after initial denaturation at 95 °C for 3 min: denaturation step at 95 °C for 30 s, annealing step at 52 °C (for *rbc*L, *mat*K, *psb*A–*trn*H) to 55 °C (for ITS) for 30–45 s, and extension step at 72 °C for 45–80 s according to product size in 35 cycles. The final extension was performed at 72 °C for 7 min. PCR results were then confirmed via high-resolution capillary electrophoresis on a QIAxcel Advanced Instrument (Qiagen, Hilden, Germany). Successfully amplified products were sequenced using an ABI 3730XL sequencer (Applied Biosystems, Foster City, CA, USA), and sequencing was performed by Macrogen (Seoul, Republic of Korea).

### 4.3. Data Analysis

Chromatogram files of sequencing were imported into Geneious R 10.2.6 (Biomatters, Auckland, New Zealand), and the nucleotide sequences were curated, analyzed, and aligned using MAFFT [74], with some parts being manually edited. All nucleotide sequences produced in this study were deposited in GenBank (Table S1, ITS: OQ150537–OQ150751, *rbc*L: OQ172533–OQ172747, *mat*K: OQ172318–OQ172532, *psb*A–*trn*H: OQ172748–OQ172962O).

The characteristics such as length, number of variable sites, and G/C content of nucleotide sequences were measured using Geneious R 10.2.6 (Biomatters), whilst the number of parsimony-informative sites was measured using PAUP 4.0b10 [75]. To evaluate DNA barcoding regions, the barcode gap was checked, which indicated whether or not interspecific and intraspecific distances overlapped. In this process, according to various combinations of the four regions, the pairwise genetic distance matrix for each individual was calculated based on the Kimura 2-parameter (K2P) method [76], which was conducted using MEGA 11 [77]. To identify species, the NJ tree was also constructed, using the K2P method throughout MEGA 11, with the bootstrap value of each node being calculated with 2000 replications. *Amaranthus spinosus* L. was used as an outgroup in this NJ analysis. (ITS: KY968964, *mat*K: MF159529, *rbc*L: MF135474, *psb*A–*trn*H: MF143791). The NJ analysis uses a distance matrix based on nucleotide differences between pairs of taxa [78], making it well-suited to DNA barcoding. Compared with other methods of phylogenetic tree construction, the NJ analysis is relatively insensitive to issues such as multiple substitutions and missing data, and it is also faster [78,79]. The success of species identification was determined by whether individuals within the species were clustered into a clade. In this process, the forma was not counted as an individual taxon. When only one individual within a species was analyzed, we decided manually by considering branch length.

Along with the NJ analysis, we used the “best close match” function in TAXONDNA to assess the success of species identification [80]. This analysis assigned a query to the species name of its best-matching barcode, regardless of the degree of similarity between the query and barcode sequences, within a distance threshold of 95% [80]. Additionally, we utilized Assemble Species by Automatic Partitioning (ASAP), an unsupervised Operational Taxonomic Unit (OTU) picking method based on pairwise sequence distance [81]. The ASAP suggests optimal species partitions with an asap-score that reflects the confidence level of the clustering. Generally, a lower score implies a better partition. This program is available online (https://bioinfo.mnhn.fr/abi/public/asap/asapweb.html (accessed on 5 May 2023)). In this process, we used default parameters, except for the substitution model (K2P method).

## Figures and Tables

**Table 1:** Statistics of four DNA barcode loci of Caryophyllaceae in Korea.

	*rbc*L	*mat*K	*psb*A–*trn*H	ITS	Total
Sequence length (bp)	689	746–782	160–404	606–637	-
Aligned length (bp)	689	784	600	672	2747
G + C ratio (%)	43.0–44.3	29.6–33.4	21.1–33.3	50.7–63.2	-
Number of variable sites (% variable sites)	124 (18.0)	431 (55.0)	398 (66.3)	393 (58.3)	1346 (49.0)
Number of informative sites (% variable sites)	115 (16.7)	411 (52.4)	371 (61.8)	381 (56.5)	1278 (46.5)

**Table 2:** Summary of the pairwise intraspecific and interspecific distances in the DNA barcode loci of Caryophyllaceae species.

Region	Intraspecific K2P Distance			Interspecific K2P Distance		
	Min.	Mean	Max.	Min.	Mean	Max.
*rbc*L	0	0.0004	0.0313	0	0.0291	0.0576
*mat*K	0	0.0019	0.1296	0	0.1140	0.1754
*psb*A–*trn*H	0	0.0078	0.4690	0	0.3245	0.7045
ITS	0	0.0037	0.2478	0	0.2259	0.3531
*rbc*L + *mat*K	0	0.0012	0.0817	0	0.0726	0.1126
*rbc*L + *psb*A–*trn*H	0	0.0022	0.1284	0	0.0888	0.1888
*rbc*L + ITS	0	0.0019	0.1246	0	0.1141	0.1657
*mat*K + *psb*A–*trn*H	0	0.0033	0.2015	0	0.1548	0.2599
*mat*K + ITS	0	0.0008	0.0072	0	0.1609	0.2340
*psb*A–*trn*H + ITS	0	0.0048	0.3077	0	0.2502	0.3754
*rbc*L + *mat*K + *psb*A–*trn*H	0	0.0021	0.1289	0	0.1003	0.1658
*rbc*L + *mat*K + ITS	0	0.0019	0.1265	0	0.1140	0.1622
*rbc*L + *psb*A–*trn*H + ITS	0	0.0028	0.1724	0	0.1405	0.2113
*mat*K + *psb*A–*trn*H + ITS	0	0.0011	0.0110	0	0.1809	0.2666
*rbc*L + *mat*K + *psb*A–*trn*H + ITS	0	0.0008	0.0077	0	0.1314	0.1936

**Table 3:** Species identification based on phylogenetic analysis and the best close match.

Region	Phylogenetic Analysis	Best Close Match			
		Correct	Ambiguous	Incorrect	No Match
*rbc*L	44.74%	84 (39.06%)	121 (56.27%)	10 (4.65%)	0 (0.00%)
*mat*K	56.58%	115 (53.48%)	74 (34.41%)	25 (11.62%)	1 (0.46%)
*psb*A–*trn*H	59.21%	139 (64.65%)	50 (23.25%)	23 (10.69%)	3 (1.39%)
ITS	76.32%	149 (69.30%)	48 (22.32%)	16 (7.44%)	2 (0.93%)
ITS + *mat*K	73.68%	160 (74.41%)	34 (15.81%)	20 (9.30%)	1 (0.46%)
ITS + *psb*A–*trn*H	76.32%	164 (76.27%)	18 (8.37%)	30 (13.95%)	3 (1.39%)
ITS + *rbc*L	77.63%	152 (70.69%)	45 (20.93%)	17 (7.90%)	1 (0.46%)
*mat*K + *psb*A–*trn*H	61.84%	141 (65.58%)	43 (20.00%)	28 (13.02%)	3 (1.39%)
*rbc*L + *mat*K	60.53%	124 (57.67%)	64 (29.76%)	27 (12.55%)	0 (0.00%)
*rbc*L *+ psb*A–*trn*H	61.84%	141 (65.58%)	44 (20.46%)	28 (13.02%)	2 (0.93%)
ITS + *mat*K + *psb*A–*trn*H	73.68%	165 (76.74%)	20 (9.30%)	28 (13.02%)	2 (0.93%)
ITS + *rbc*L + *mat*K	73.68%	163 (75.81%)	28 (13.02%)	23 (10.69%)	1 (0.46%)
ITS + *rbc*L + *psb*A–*trn*H	76.32%	164 (76.27%)	17 (7.90%)	32 (14.88%)	2 (0.93%)
*rbc*L *+ mat*K + *psb*A–*trn*H	59.21%	146 (67.9%)	32 (14.88%)	36 (16.74%)	1 (0.46%)
ITS + *rbc*L + *mat*K + *psb*A–*trn*H	75.00%	165 (76.74%)	18 (8.37%)	31 (14.41%)	1 (0.46%)

**Table 4:** List of primers for the four regions sequenced in this study.

Region	Primer Name	Primer Sequence (′5-′3)	Reference
ITS	ITS18S-A	CCTTMTCATYTAGAGGAAGGAG	Muir & Schlötterer [69]
	ITS4	TCCTCCGCTTATTGATATGC	White et al. [70]
*rbc*L	rbcL 1F	ATGTCACCACAAACAGAAAC	Fay et al. [71]
	rbcL 724R	TCGCATGTACCTGCAGTAGC	
*mat*K	matK_3F	CGTACAGTACTTTTGTGTTTACGAG	Kim K.J. (unpublished work)
	matK_1R	ACCCAGTCCATCTGGAAATCTTGGTTC	
*psb*A–*trn*H	psbA_sang	GTTATGCATGAACGTAATGCTC	Sang et al. [72]
	trnH_tate	CGCGCATGGTGGATTCACAATCC	Tate [73]

## Data Availability

The four chloroplast genomes, newly sequenced in this study, were archived in NCBI with accession numbers OQ150537–OQ150751 and OQ172318–OQ172962.

## Supplementary Materials

The following supporting information can be downloaded at: https://www.mdpi.com/article/10.3390/plants12102060/s1, Figure S1: Species partitions through ASAP algorithm based on *rbc*L; Figure S2: Species partitions through ASAP algorithm based on *mat*K; Figure S3: Species partitions through ASAP algorithm based on *psb*A–*trn*H; Figure S4: Species partitions through ASAP algorithm based on internal transcribed spacer (ITS); Figure S5: Species partitions through ASAP algorithm based on ITS + *mat*K; Figure S6: Species partitions through ASAP algorithm based on ITS + *psb*A–*trn*H; Figure S7: Species partitions through ASAP algorithm based on ITS + *rbc*L; Figure S8: Species partitions through ASAP algorithm based on *mat*K + *psb*A–*trn*H; Figure S9: Species partitions through ASAP algorithm based on *mat*K + *rbc*L; Figure S10: Species partitions through ASAP algorithm based on *psb*A–*trn*H + *rbc*L; Figure S11: Species partitions through ASAP algorithm based on ITS + *mat*K + *rbc*L; Figure S12: Species partitions through ASAP algorithm based on ITS + *psb*A–*trn*H + *rbc*L; Figure S13: Species partitions through ASAP algorithm based on *rbc*L + *psb*A–*trn*H + *rbc*L; Figure S14: Species partitions through ASAP algorithm based on ITS + *mat*K + *psb*A–*trn*H + *rbc*L; Figure S15: The neighbor-joining phylogenetic tree of the Korean Caryophyllaceae based on combined sequence from ITS + *psb*A–*trn*H; Table S1: List of species sequenced in this study and GenBank accession number.
