# Supplementary material for: Identification of the Plant Family Caryophyllaceae in Korea Using DNA Barcoding
Source: Plants (Basel). 2023 May 22;12(10):2060. doi: 10.3390/plants12102060 (PMC10222892; doi:10.3390/plants12102060)
Supplement: Supplementary file 1 [file plants-12-02060-s001.zip › plants-2295811-supplementary.pdf]

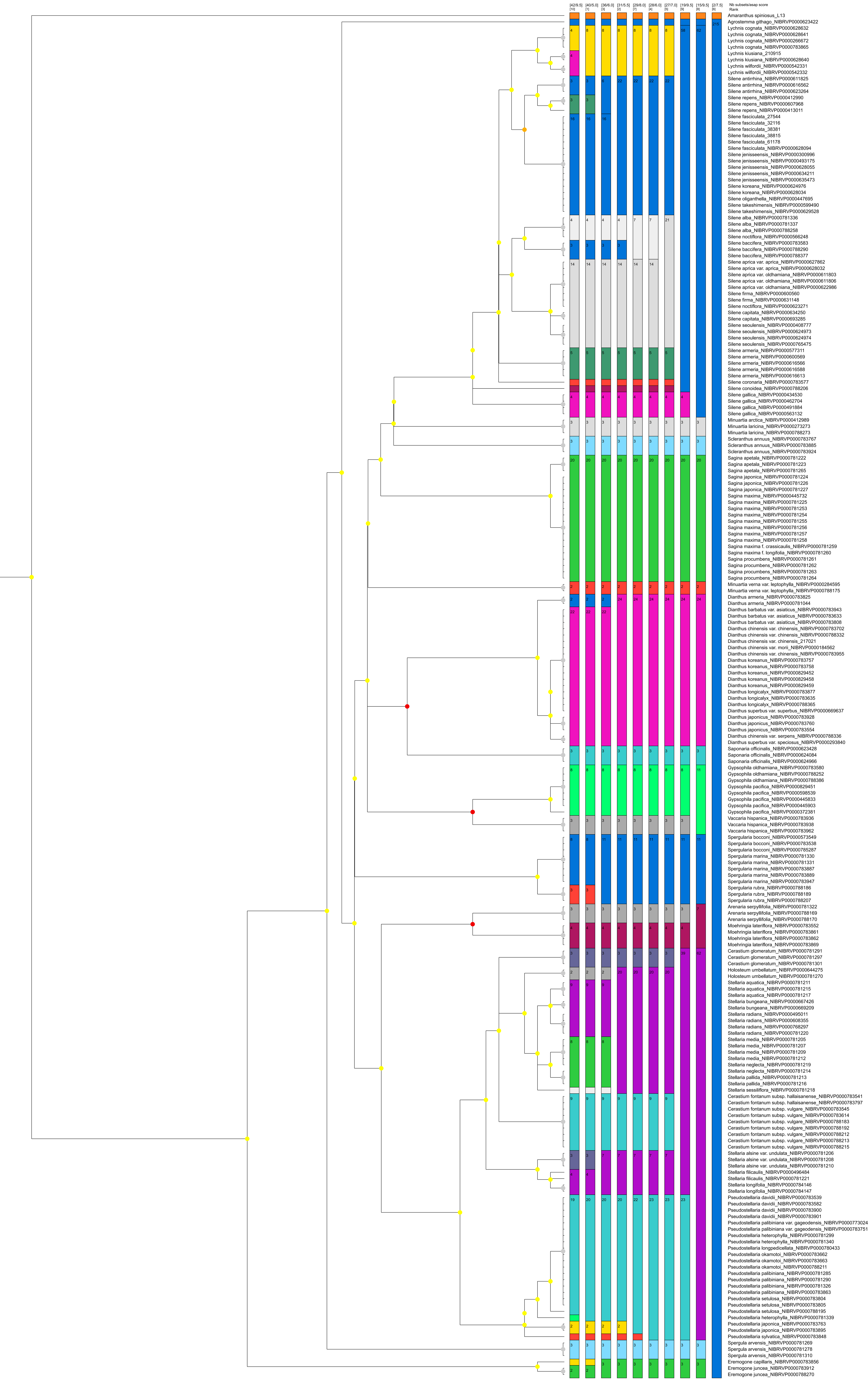

Figure S1. Species partitions through ASAP algorithm based on *rbc*L.

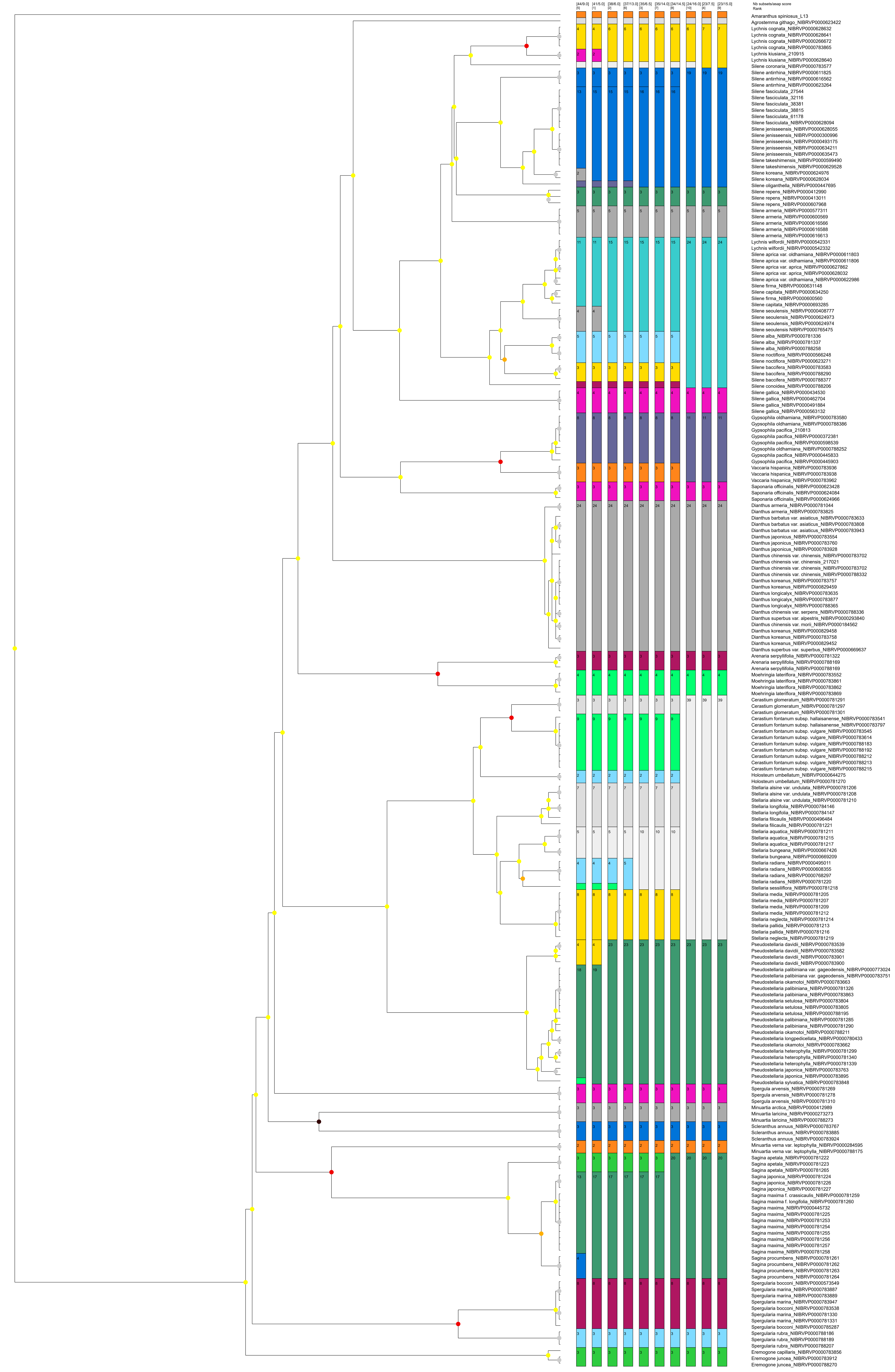

Figure S2. Species partitions through ASAP algorithm based on *mat*K.

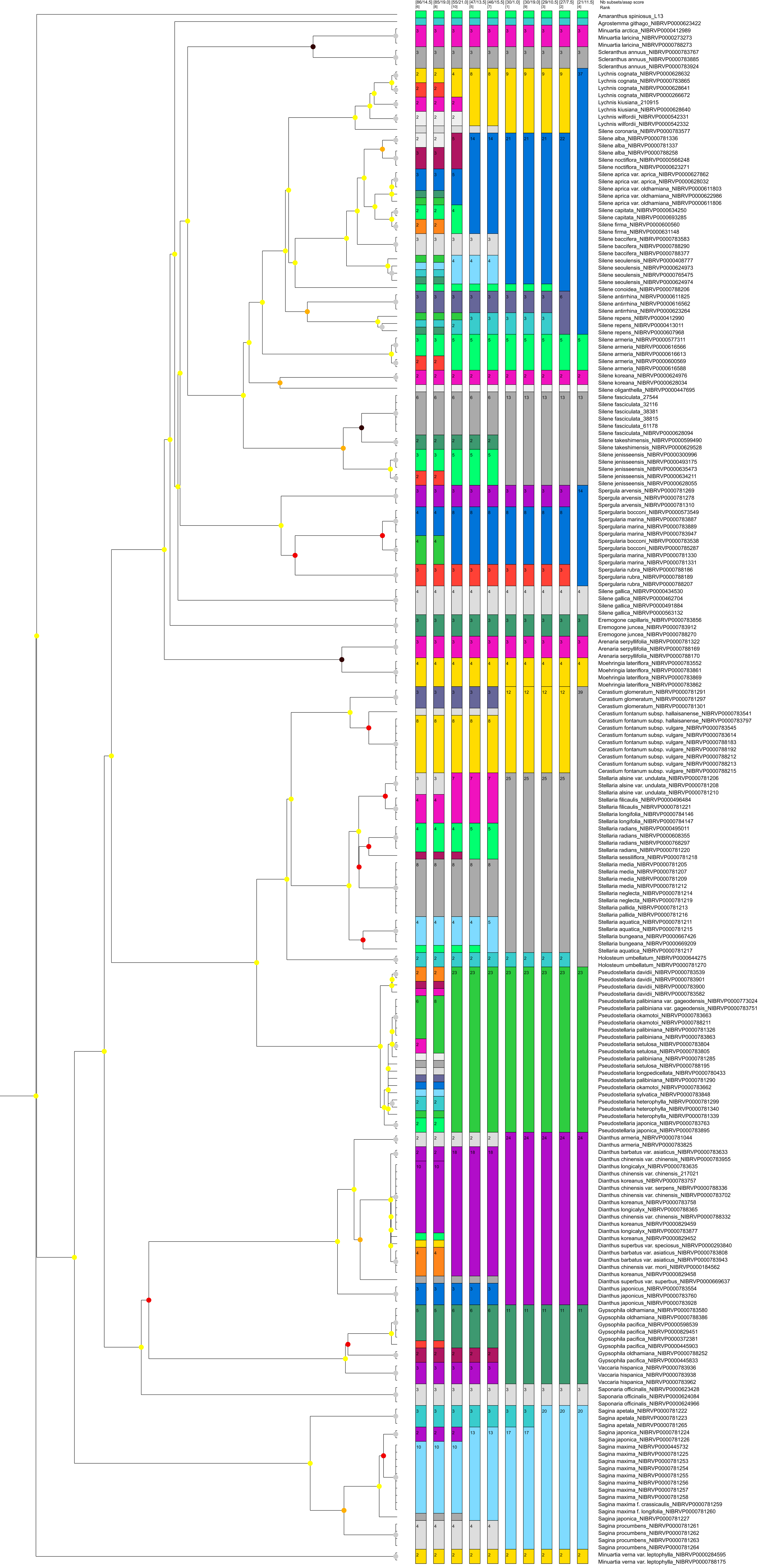

Figure S3. Species partitions through ASAP algorithm based on *psb*A–*trn*H.

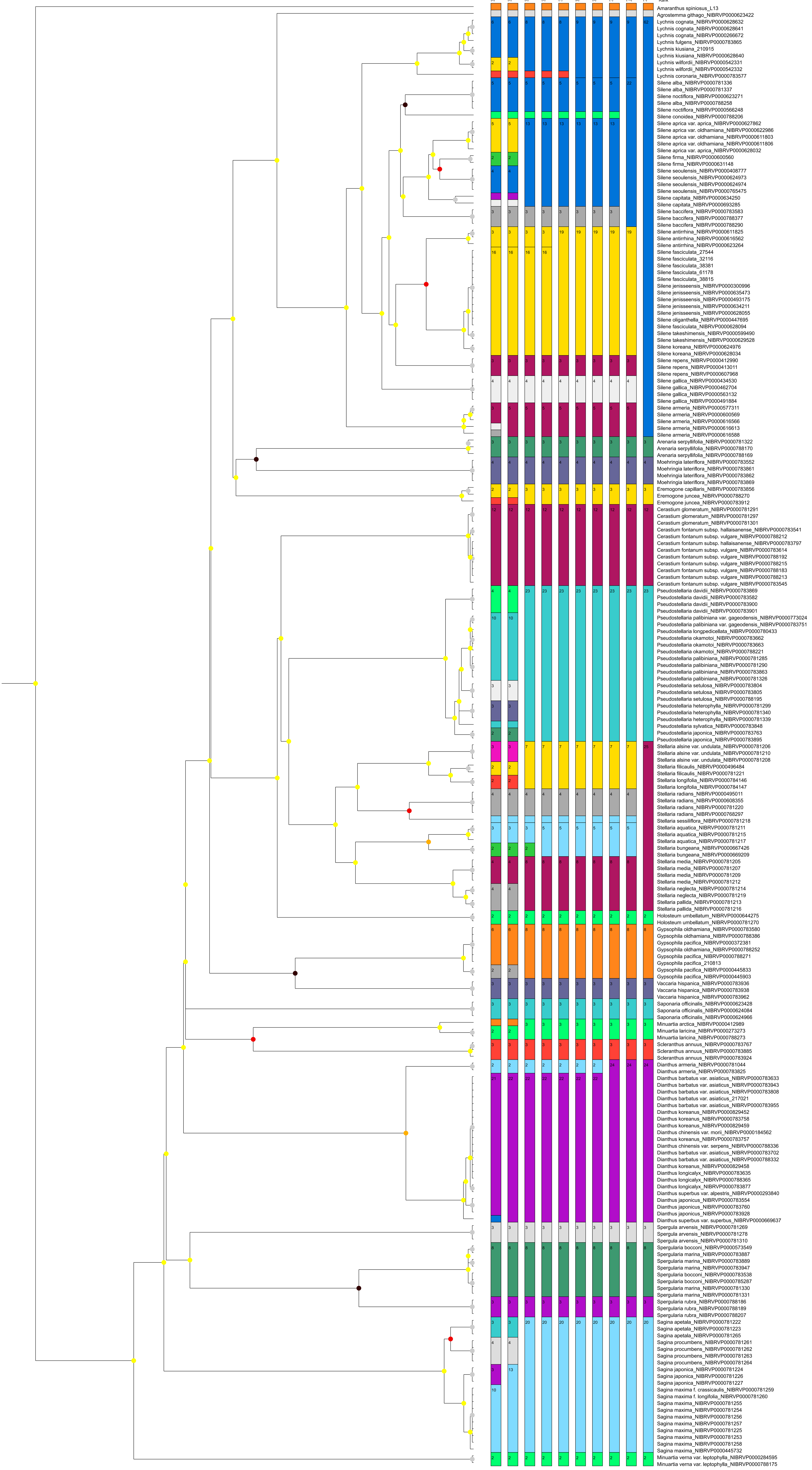

Figure S4. Species partitions through ASAP algorithm based on internal transcribed spacer (ITS).

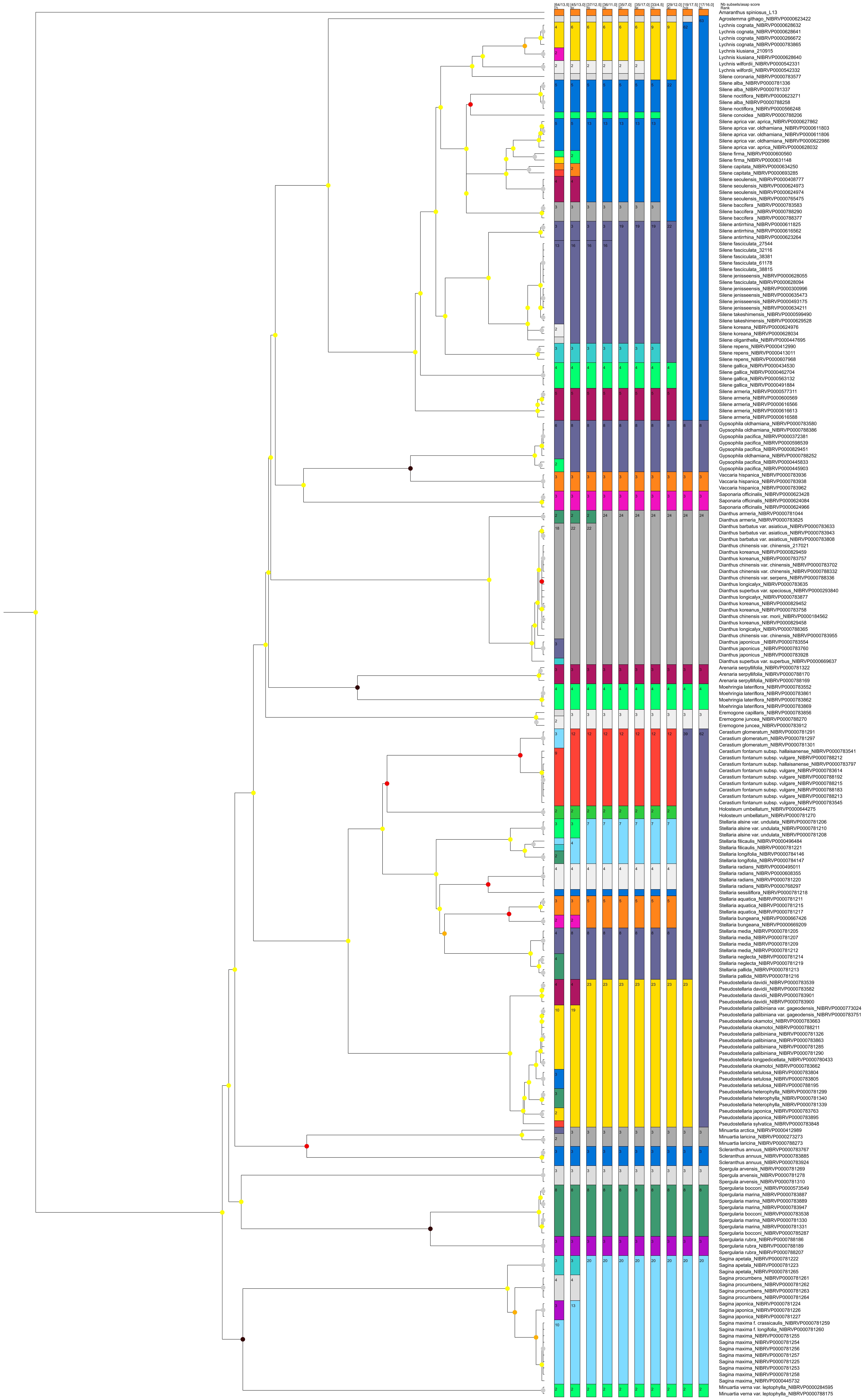

Figure S5. Species partitions through ASAP algorithm based on ITS + *mat*K.

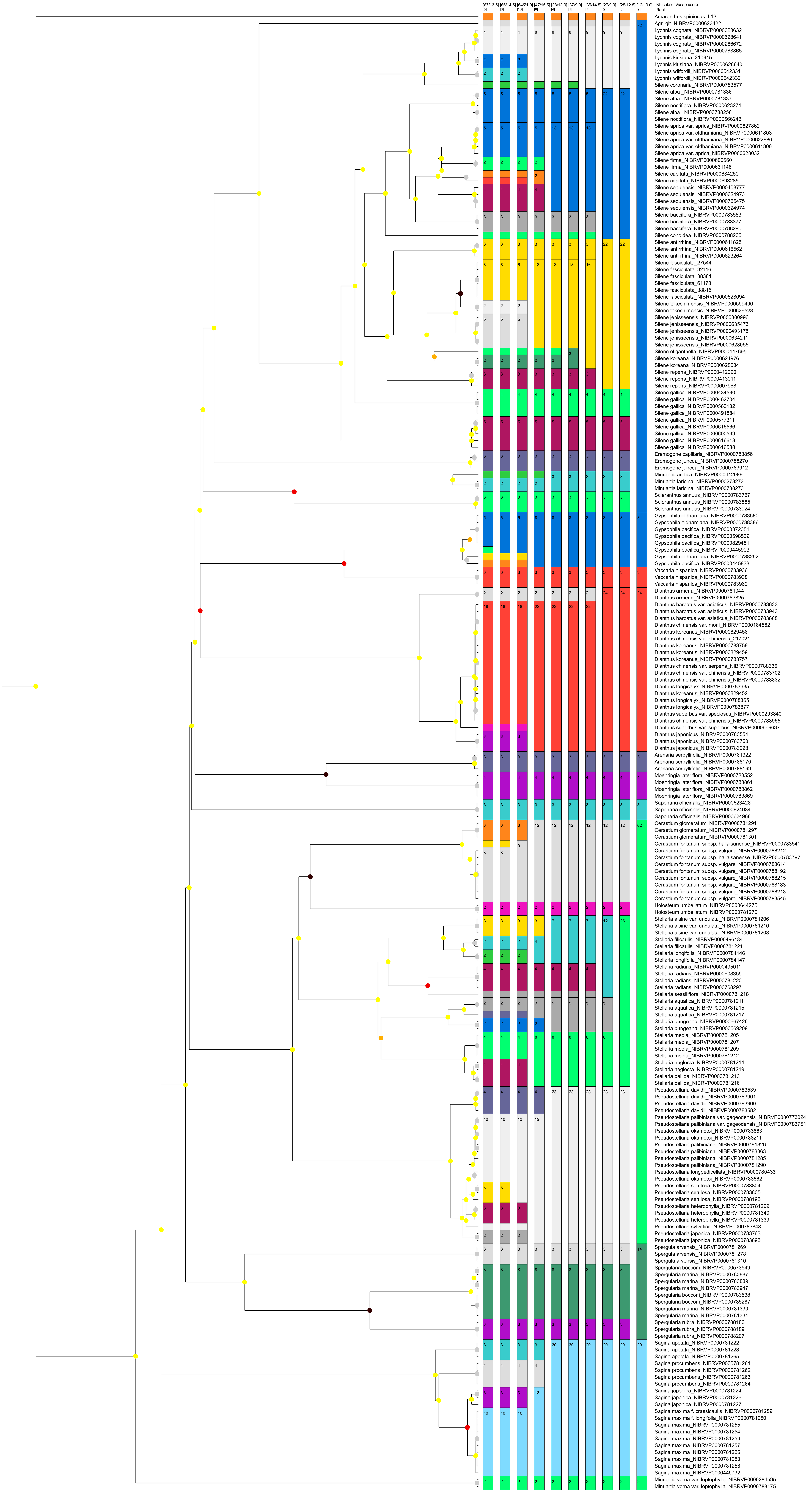

Figure S6. Species partitions through ASAP algorithm based on ITS + *psb*A–*trn*H.

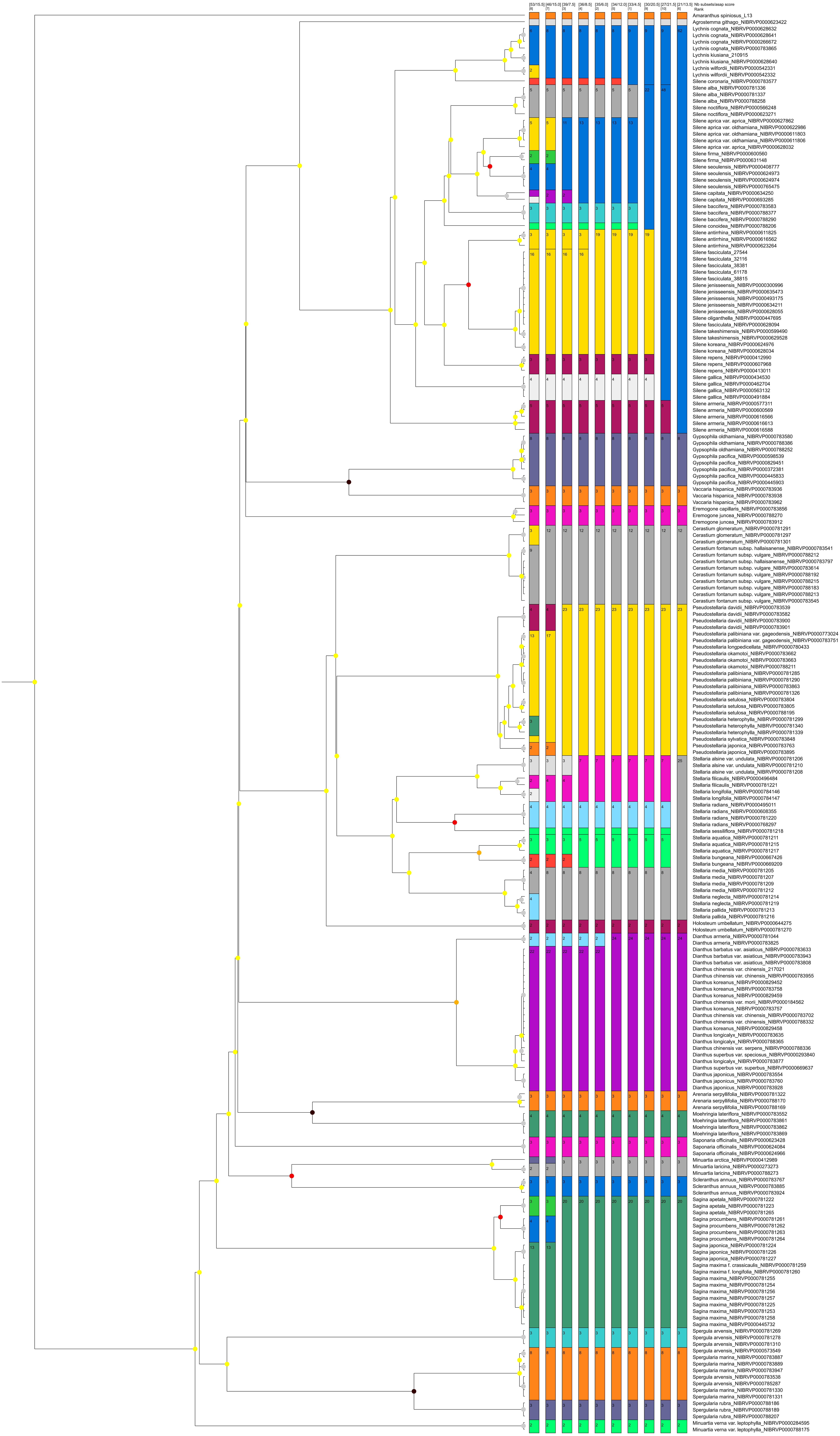

Figure S7. Species partitions through ASAP algorithm based on ITS + *rbc*L.

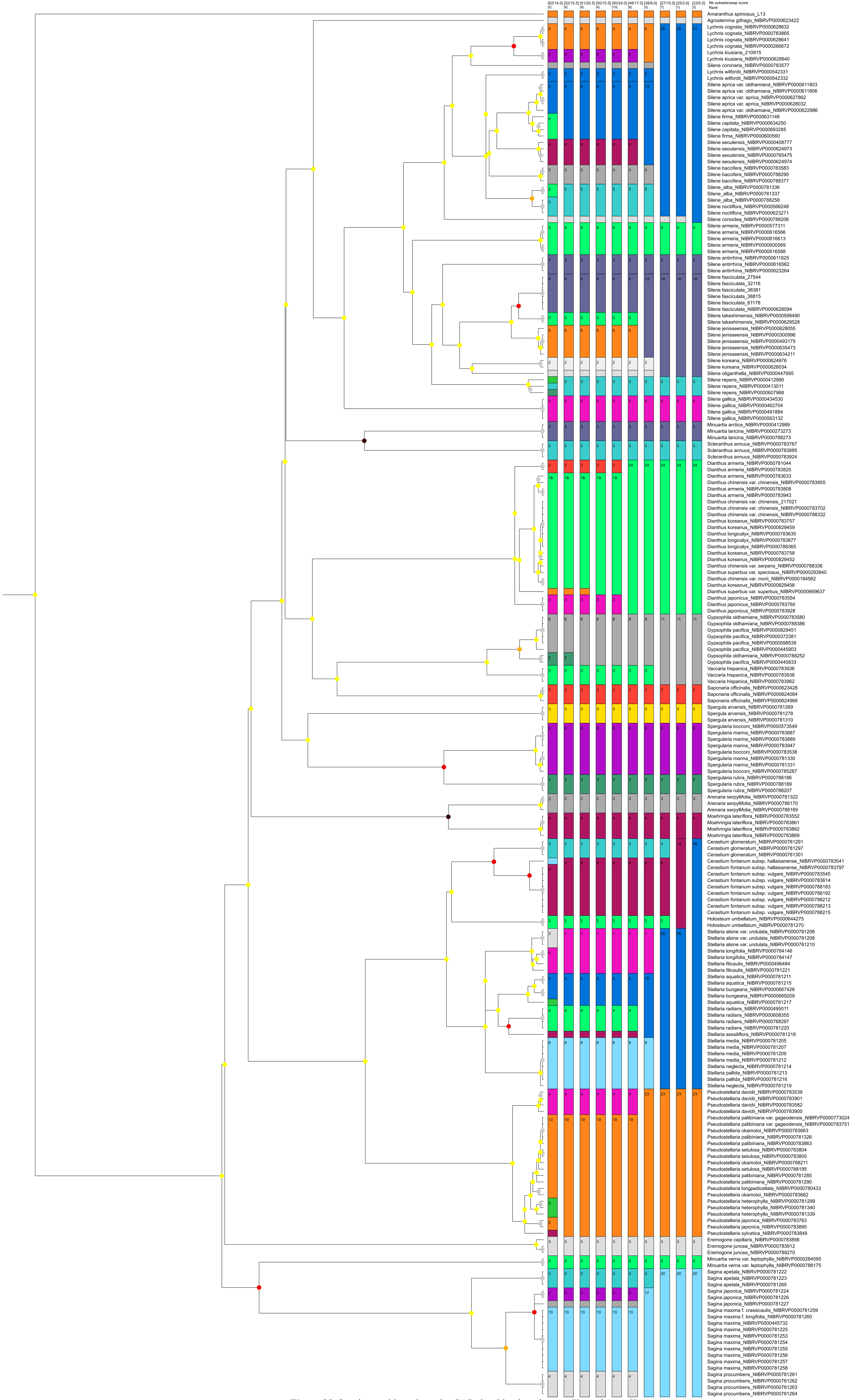

Figure S8. Species partitions through ASAP algorithm based on *mat*K + *psb*A–*trn*H.

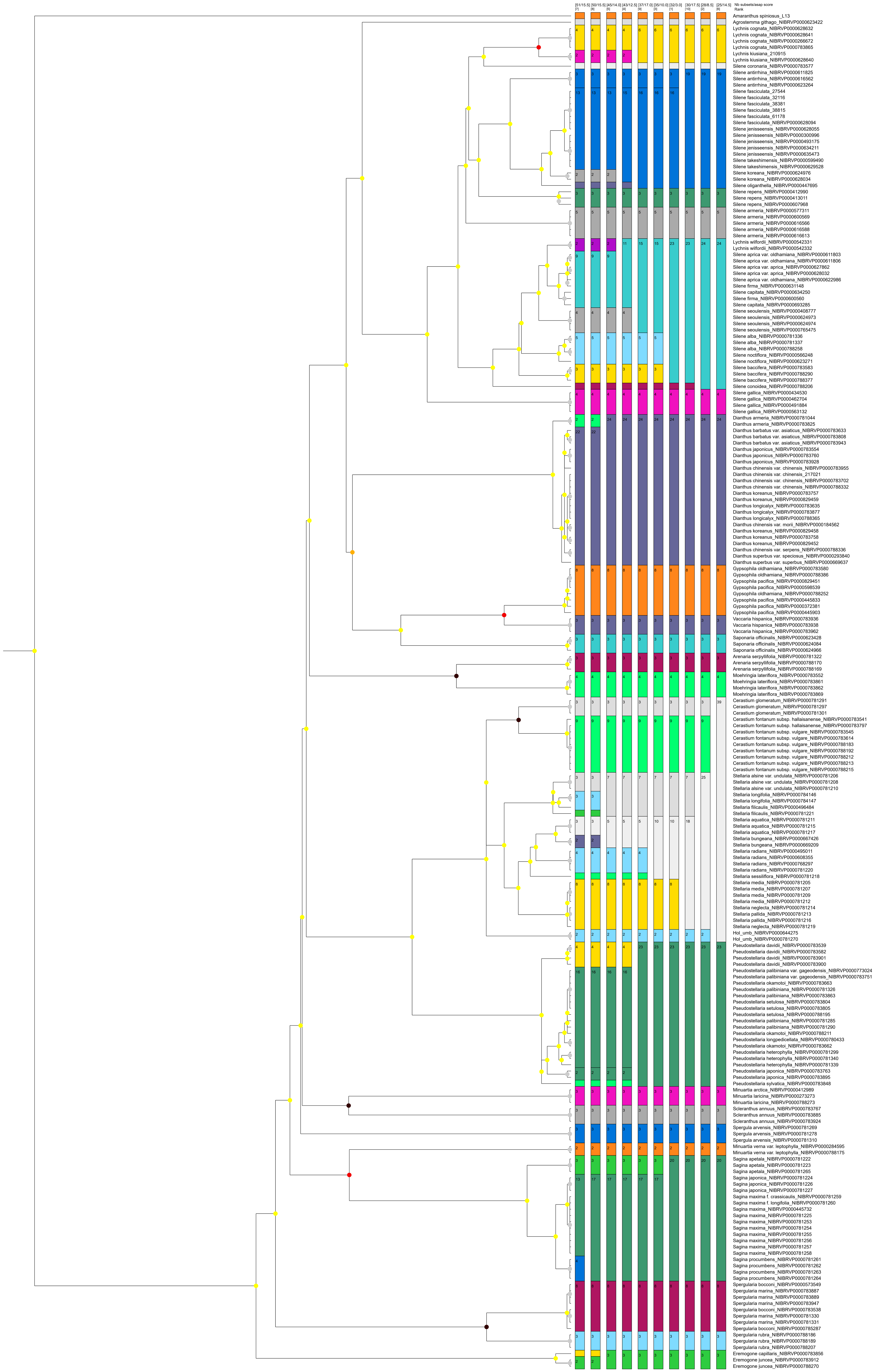

Figure S9. Species partitions through ASAP algorithm based on *mat*K + *rbc*L.

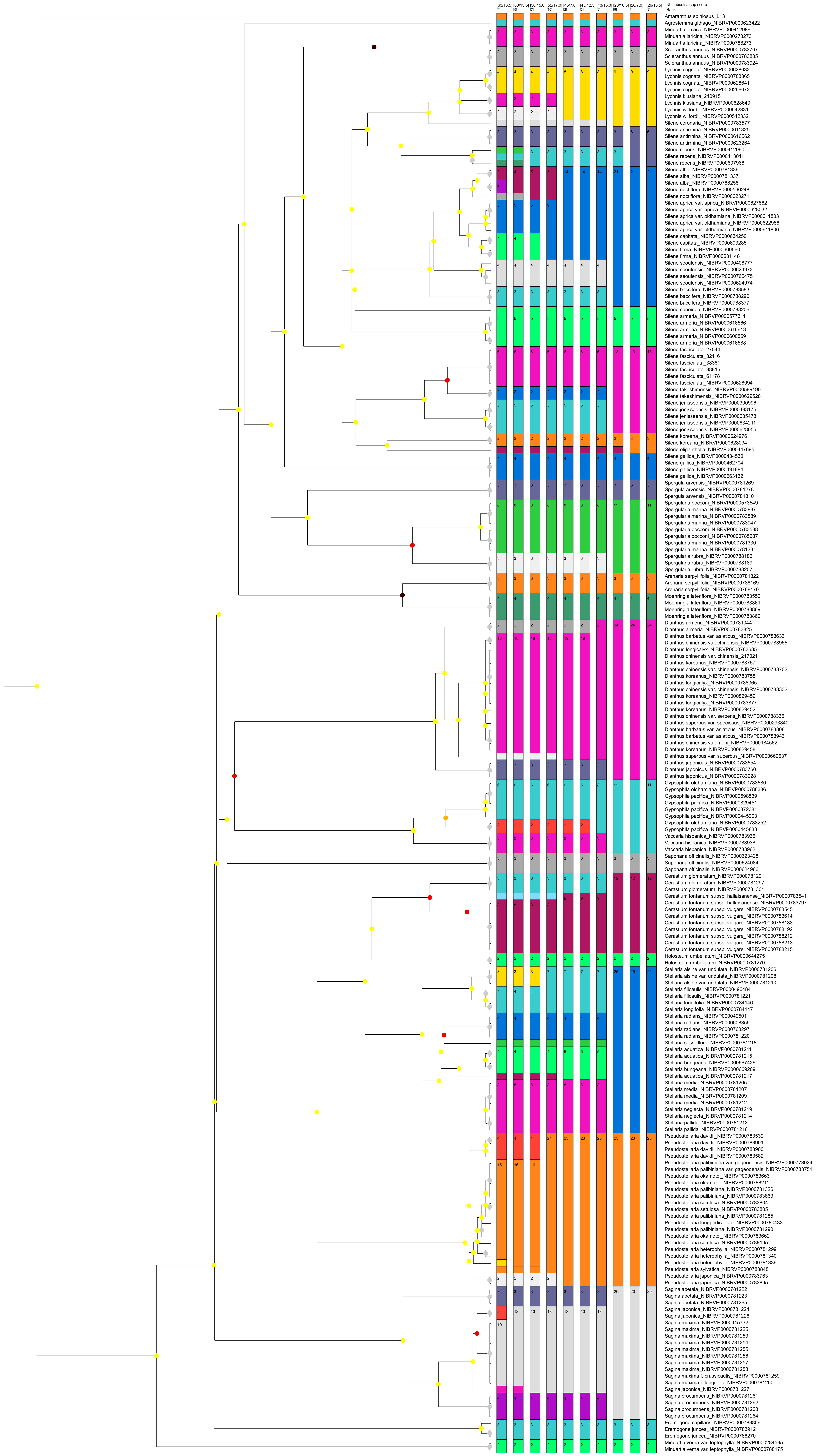

Figure S10. Species partitions through ASAP algorithm based on *psb*A–*trn*H + *rbc*L.

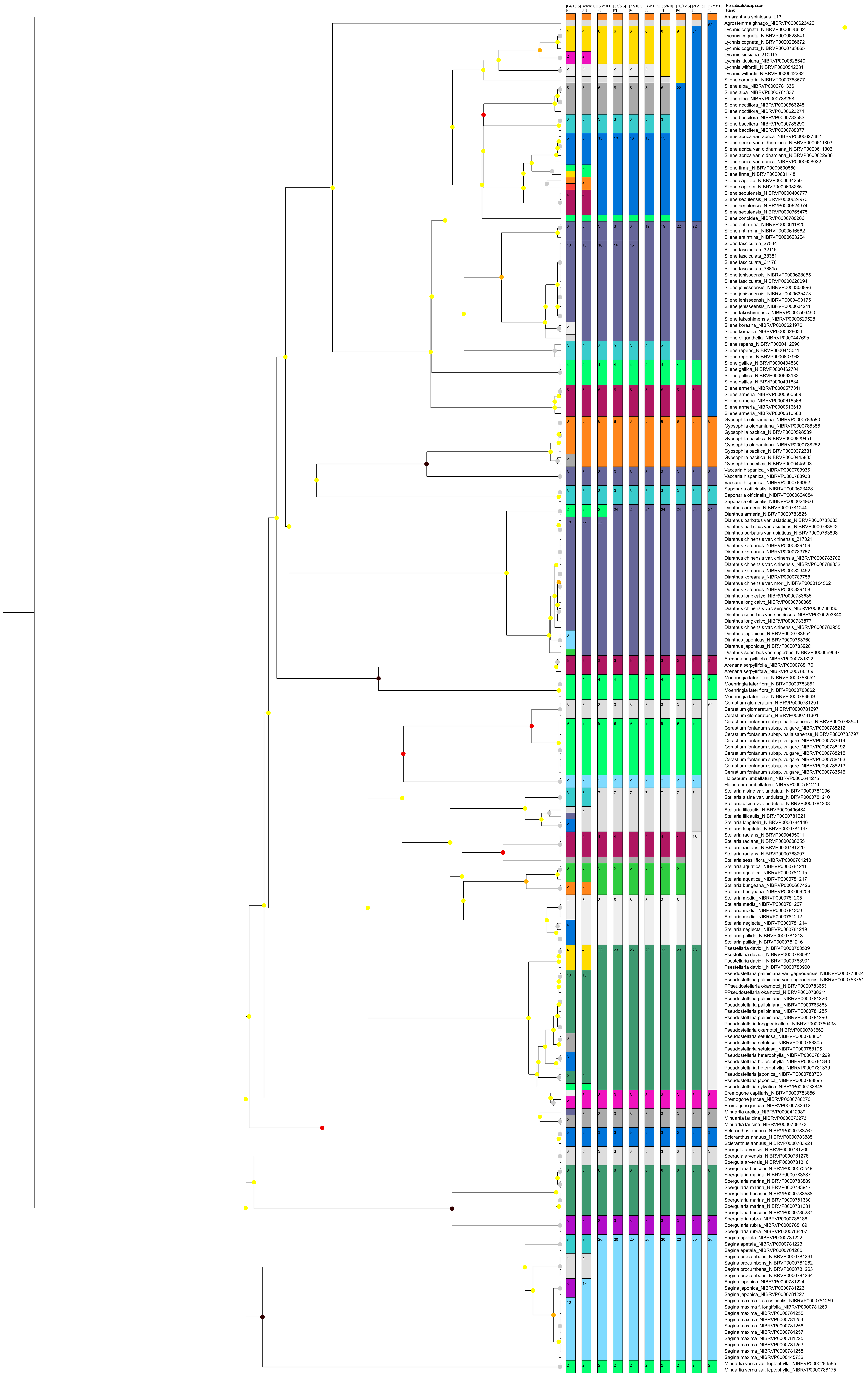

Figure S11. Species partitions through ASAP algorithm based on ITS + *mat*K + *rbc*L.

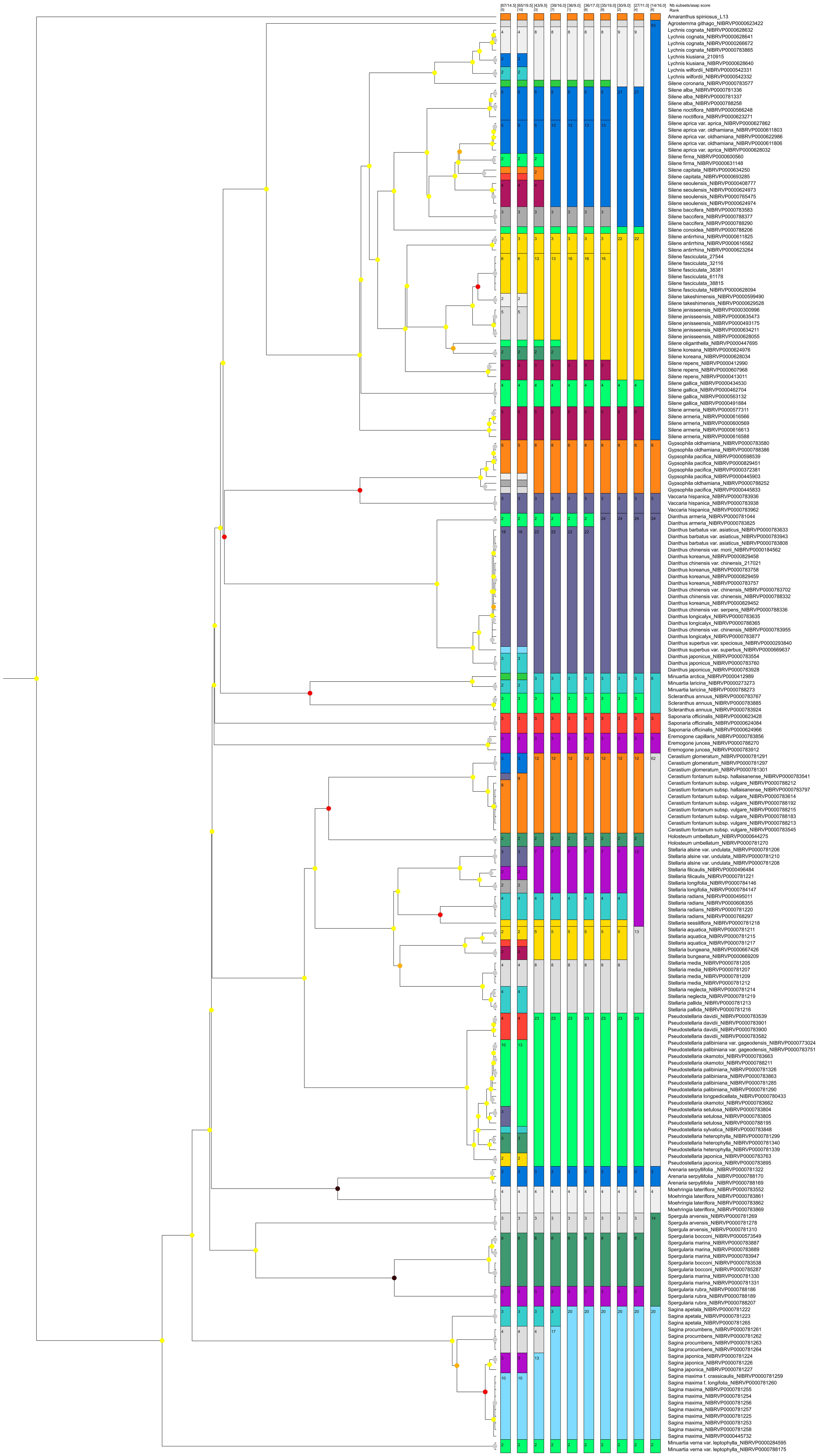

Figure S12. Species partitions through ASAP algorithm based on ITS + *psb*A–*trn*H + *rbc*L.

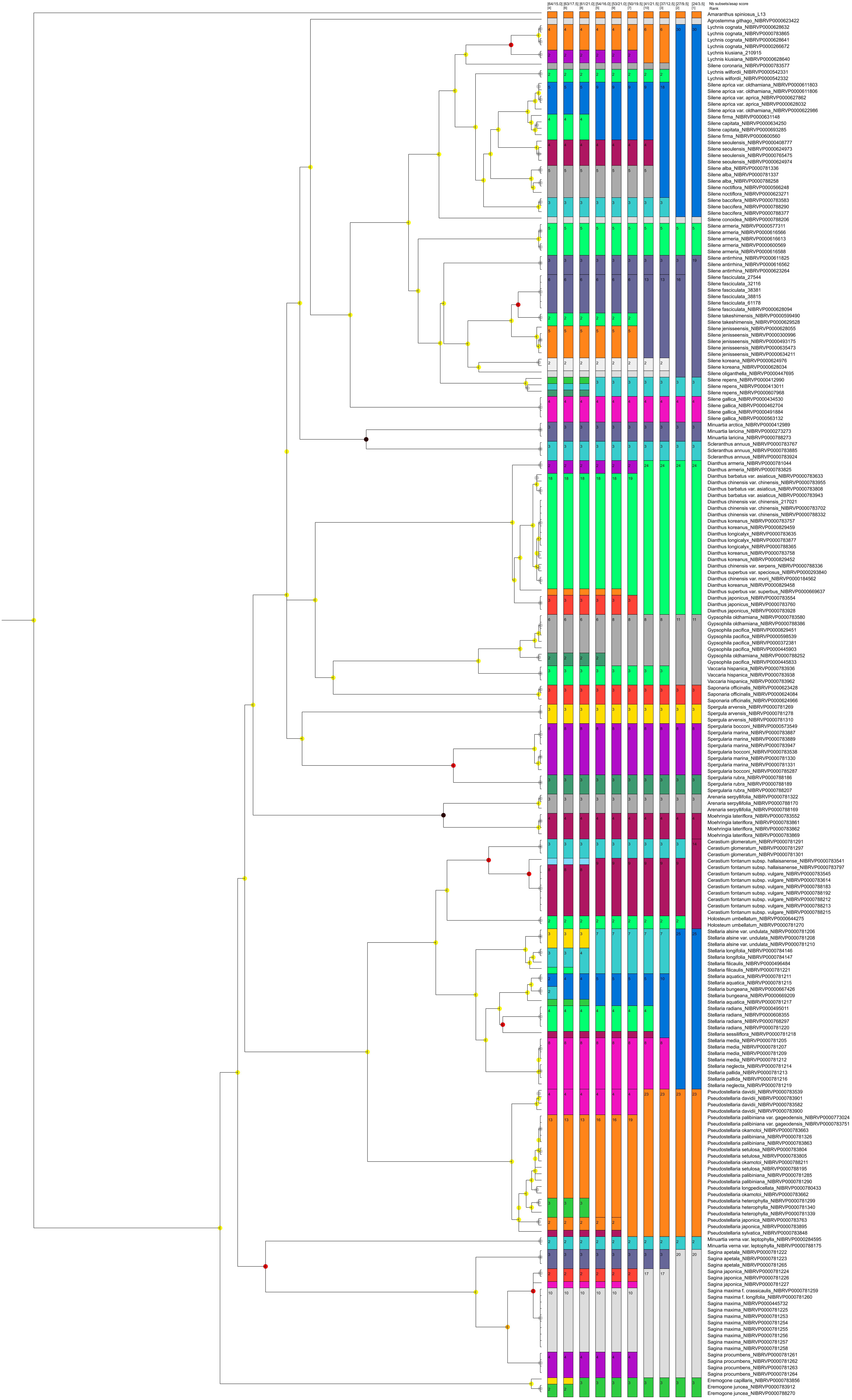

Figure S13. Species partitions through ASAP algorithm based on *rbc*L + *psb*A–*trn*H + *rbc*L.

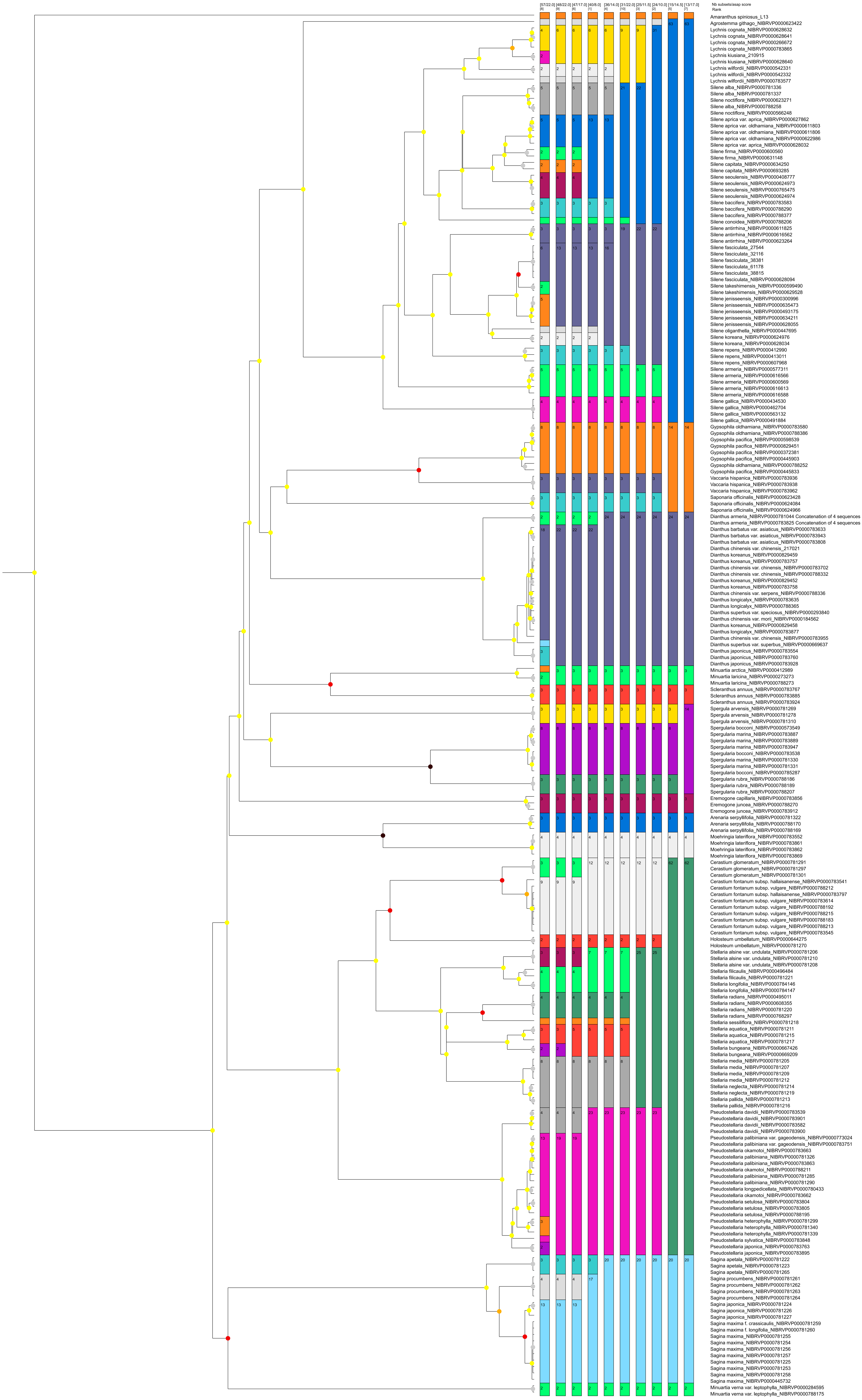

Figure S14. Species partitions through ASAP algorithm based on ITS + *mat*K + *psb*A–*trn*H + *rbc*L.

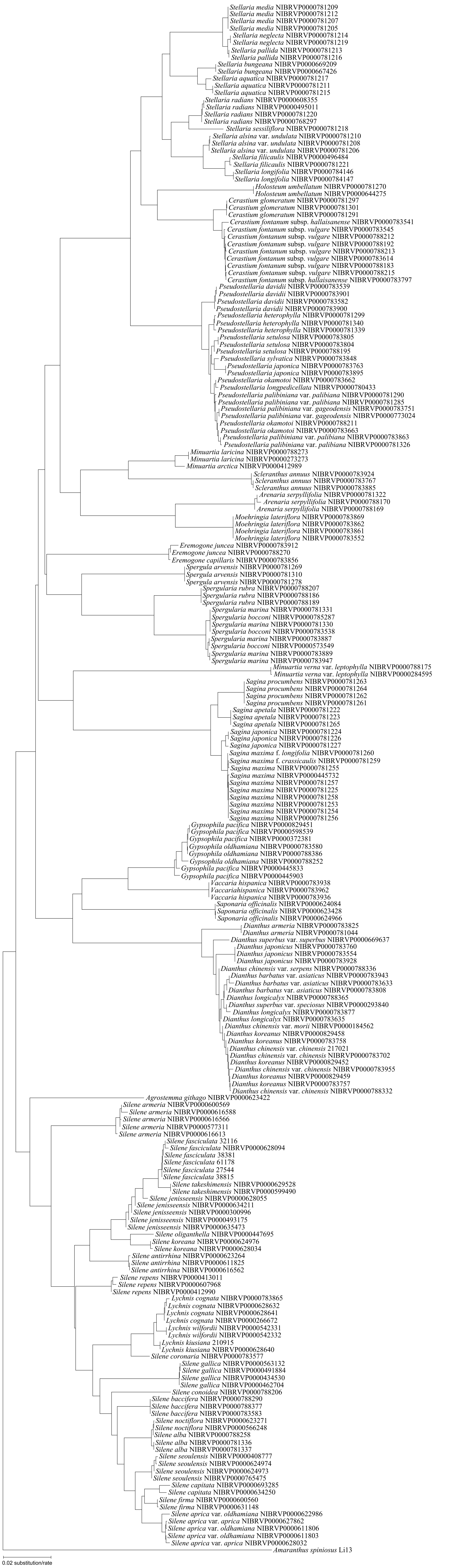

Figure S15. The neighbor-joining phylogenetic tree of the Korean Caryophyllaceae based on combined sequence from ITS + *psb*A–*trn*H.
